# Dissecting miRNA–Gene Networks to Map Clinical Utility Roads of Pharmacogenomics-Guided Therapeutic Decisions in Cardiovascular Precision Medicine

**DOI:** 10.3390/cells11040607

**Published:** 2022-02-10

**Authors:** Fani Chatzopoulou, Konstantinos A. Kyritsis, Christos I. Papagiannopoulos, Eleftheria Galatou, Nikolaos Mittas, Nikoleta F. Theodoroula, Andreas S. Papazoglou, Efstratios Karagiannidis, Maria Chatzidimitriou, Anna Papa, Georgios Sianos, Lefteris Angelis, Dimitrios Chatzidimitriou, Ioannis S. Vizirianakis

**Affiliations:** 1 Laboratory of Microbiology, School of Medicine, Aristotle University of Thessaloniki, 54124 Thessaloniki, Greece; fchatzop@auth.gr (F.C.); annap@auth.gr (A.P.); dihi@auth.gr (D.C.); 2 Labnet Laboratories, Department of Molecular Biology and Genetics, 54638 Thessaloniki, Greece; 3 Laboratory of Pharmacology, School of Pharmacy, Aristotle University of Thessaloniki, 54124 Thessaloniki, Greece; kkyritsis@pharm.auth.gr (K.A.K.); papagiac@pharm.auth.gr (C.I.P.); theodorn@pharm.auth.gr (N.F.T.); 4 Department of Life & Health Sciences, University of Nicosia, Nicosia 1700, Cyprus; galatou.e@unic.ac.cy; 5 Department of Chemistry, International Hellenic University, 65404 Kavala, Greece; nmittas@chem.ihu.gr; 6 1st Cardiology Department, AHEPA University General Hospital of Thessaloniki, 54636 Thessaloniki, Greece; anpapazoglou@yahoo.com (A.S.P.); stratoskarag@gmail.com (E.K.); gsianos@auth.gr (G.S.); 7 Department of Biomedical Sciences, School of Health Sciences, International Hellenic University, 57400 Thessaloniki, Greece; mchatzid952@gmail.com; 8 Department of Informatics, Aristotle University of Thessaloniki, 54124 Thessaloniki, Greece; lef@csd.auth.gr

**Keywords:** miRNAs, biomarkers, gene networks, cardiovascular disorders, precision medicine, pharmacogenomics, SNPs, clinical trials

## Abstract

MicroRNAs (miRNAs) create systems networks and gene-expression circuits through molecular signaling and cell interactions that contribute to health imbalance and the emergence of cardiovascular disorders (CVDs). Because the clinical phenotypes of CVD patients present a diversity in their pathophysiology and heterogeneity at the molecular level, it is essential to establish genomic signatures to delineate multifactorial correlations, and to unveil the variability seen in therapeutic intervention outcomes. The clinically validated miRNA biomarkers, along with the relevant SNPs identified, have to be suitably implemented in the clinical setting in order to enhance patient stratification capacity, to contribute to a better understanding of the underlying pathophysiological mechanisms, to guide the selection of innovative therapeutic schemes, and to identify innovative drugs and delivery systems. In this article, the miRNA–gene networks and the genomic signatures resulting from the SNPs will be analyzed as a method of highlighting specific gene-signaling circuits as sources of molecular knowledge which is relevant to CVDs. In concordance with this concept, and as a case study, the design of the clinical trial GESS (NCT03150680) is referenced. The latter is presented in a manner to provide a direction for the improvement of the implementation of pharmacogenomics and precision cardiovascular medicine trials.

## 1. Introduction

Cardiovascular disorders (CVDs) represent a group of diseases which are associated with a large number of risk factors—including environmental, genomics and lifestyle risks—that contribute to both development and disease progression. CVDs are responsible for most noncommunicable disease (NCD) deaths, the number of which increased dramatically over the last two decades. According to the latest report from the World Health Organization (WHO) in 2019, NCDs accounted for 74% of all deaths globally (about 41 million people), while CVDs account for about 17.9 million deaths annually, for an estimated 32% of all deaths globally. Moreover, CVDs including coronary artery disease (CAD), myocardial infarction (MI), heart failure (HF) and cardiomyopathies rank among the leading causes of morbidity and mortality worldwide [1].

After the completion of the Human Genome Project, the efforts focusing on the clinical translation of the extracted molecular knowledge strengthened personalized medicine concepts to emerge and be applied in the healthcare system. Developments are currently being witnessed in the “-omics” methodologies, nanotechnological approaches and artificial intelligence capabilities, which now present greater accuracy, safety, and efficiency in the facilitation of disease prognosis and diagnosis, as well as the delivery of therapeutics in the clinical setting for specific populations and even individual patients [2,3]. In particular, for diseases and disorders exhibiting a complex and multifactorial status in their pathophysiology, like CVDs, such a capacity advances the improvement of clinical outcomes and offers more cost-effective disease management. Importantly, the clinical translation of the molecular knowledge has to be suitably implemented within existing therapeutic intervention guidelines, as well as laboratory tests and specific patient group handling procedures and protocols [4]. In order to move towards this direction, the molecular background reflecting the cardiovascular system’s physiology and pathophysiology must be clearly understood.

The genetic nature of CVDs is polygenetic, with a plethora of genes being involved in various signaling pathways that affect vital cellular decisions. Although the latter complicates a universal and easy exploitation of the genetic information, the validation of the clinical practical utility of specific identified biomarkers coming from genomic studies (e.g., single nucleotide polymorphisms (SNPs), differential gene and protein expression levels, and the identification of specific RNA molecules) offers such a capability. In previous years, evidence has shown that multiple such biomarkers often contribute cumulatively and generate polygenic risk scores that reflect an individual’s susceptibility to the emergence of disease before any symptoms appear. Moreover, by using predictive biomarkers for the early diagnosis of CVDs, medications may be delivered earlier to patients in order to ameliorate their risk and improve their clinical outcomes. Indeed, the administration of cardiovascular drugs offered in the clinical setting is based on pharmacological targets controlling the symptoms and thus minimizing the contribution of specific risk factors to CVDs’ emergence (e.g., high pressure and high cholesterol levels) [5,6,7,8,9].

The pathophysiology of CVDs is heterogeneous and involves various risk factors that contribute to the development and disease progression, whereas interindividual genetic differences further contribute to illness complexity and severity seen in the clinical phenotype”. Therefore, it is vital to analyze and clinically handle the extracted and validated genomic knowledge in a way which enables the identification of the most common molecular pathways derived from prognostic, diagnostic and pharmacogenomics biomarkers. MiRNAs represent crucial players at the molecular level that epigenetically modulate gene expression and function at the cellular and organismal level. Because miRNAs can target multiple genes, they can be valuable biomarkers in the management of various CVDs. In this context, the clinical utility of validated miRNA biomarkers in CVDs will be discussed by analyzing the miRNA-gene networks and correlating the molecular pathophysiology with pharmacogenomics knowledge. In particular, this review highlights the following for CVDs: (1) the role of miRNAs and their value in precision medicine, with special emphasis being given to atherosclerosis (arteriosclerosis); (2) the need for the implementation of pharmacogenomics to advance precision in CVD diagnosis and prognosis; (3) the power of biomarker miRNAs to advance pharmacogenomics knowledge by linking their gene networks with pathophysiology signaling pathways, SNPs, and drug targets. By combining the gene pharmacological targets of the marketed drugs used for the therapeutic management of CVDs within the miRNA–gene networks, the utility of different biomarkers (miRNAs, SNPs, pharmacogenomic) can be pinpointed across the pathway in order to guide clinical decisions. To this end, in silico methodologies, machine learning approaches and data-driven predictive risk models are needed to permit the validation and implementation of miRNA biomarkers in the care of CVDs for individual patients and populations.

The implementation of microRNAs and other biomarkers in the clinical setting and therapeutics of CVDs has been thoroughly covered in recently published reviews [10,11,12,13,14,15]. Moreover, the attempt to identify circulating microRNAs as biomarkers and the elucidation of the role of exosomes in carrying such critical molecules in the plasma of patients with CDVs has been additionally shown to present promises in diagnosis, therapy, and drug delivery [16,17,18,19]. To this end, the emerging role of circular RNAs (circRNAs) as potential biomarkers in CVDs has also been highlighted [20,21]. Furthermore, as recently pointed out, the long noncoding RNAs (lncRNAs) that represent a heterogenous group of noncoding transcripts are capable of regulating complex molecular networks, and are therefore considered as potential biomarkers in CVDs [22,23]. The main focus of our work relies on the development and application of bioinformatic methodologies capable to uncover pharmacogenomics relevant biomarkers in the miRNAs-gene correlation axis to guide cardiovascular medicine decisions. This is crucial as there are various types of genetic traits, (e.g., expression and mutational level, SNPs, epigenetic modifications, drug targets) within miRNA-gene networks that must be assessed and applied simultaneously in the clinical setting as biomarkers. Thus, the data presented in this article provides new insights into bioinformatics analysis of miRNA-gene interactions by presenting approaches to better achieve biomarker implementation for the diagnosis, treatment, and development of new drugs in CVDs. Overall, such direction contributes to efforts aiming to emphasize the importance of clinically-relevant correlations in the miRNA-genes axis that exhibit an importance to advance pharmacogenomics and cardiovascular precision medicine.

## 2. Role of miRNAs in Cardiovascular System Physiology and Pathophysiology

Atherosclerosis is a progressive, chronic, inflammatory disease of the artery walls caused by endothelial damage or injury. Blood cells and other substances such as fats, cholesterol and matrix proteins clump at the injury site, initiating atherosclerotic plaque formation and subsequent vessel stenosis. Although the exact cause of the plaque formation is still unclear, hypertension, hypercholesterolemia, diabetes, obesity, and smoking are considered to be major risk factors. Atherosclerosis is the main underlying cause of a plethora of CVDs, such as CAD, MI, stroke, carotid artery disease, and peripheral vascular disease.

Despite the significant advances in cardiovascular research, the current prognostic, diagnostic, and therapeutic tools have not been adequately improved, and fail to manage patients suffering from various CVDs. Thus, the need for the development of novel accurate prognostic and therapeutic tools remains a major challenge in cardiovascular medicine. Over the past few years, human genome sequencing studies have improved the characterization of non-coding RNAs, and have revealed their important roles in health and the pathophysiological pathways of many diseases, including cancer, diabetes, and CVDs [24,25]. Based on size, non-coding RNAs can be categorized into short non-coding RNAs (sncRNAs < 200 nucleotides) and long non-coding RNAs (lncRNAs > 200 nucleotides up to 100 kilobases). According to their function and structure, sncRNAs can be divided into: (a) functional RNAs, which play pivotal roles in transcription and translation, such as transfer-RNAs (t-RNAs), small nuclear RNAs (snRNAs), and ribosomal RNAs (rRNAs); and (b) regulatory RNAs, which differentially regulate gene expression, such as microRNAs (miRNAs), small interfering RNAs (siRNAs), and piwi-interacting RNAs (piwiRNAs) [26,27,28].

Among this wide variety of non-coding RNAs, much attention has been given to miRNAs which consist of 20–22 nucleotides, as they play key regulatory roles for molecular networks of cardiac development, lipid homeostasis and CVD pathogenesis [29,30]. MiRNAs regulate the expression of protein-coding genes through post-transcription mechanisms, mainly by inhibiting the translation of target mRNAs or inducing their destabilization. Specifically, a single miRNA may bind to complementary sequences of multiple target mRNAs, and a unique mRNA can be the target of several miRNAs, thus forming large-scale miRNA-mRNA regulatory networks. Besides their role in posttranscriptional regulation, miRNAs exert epigenetic regulatory roles [31].

Through their role in the regulation of gene expression, miRNAs can regulate the drug response by acting on genes encoding metabolic enzymes, protein carriers and pharmacological targets. At the same time, the presence of many miRNAs in the bloodstream has sparked a wealth of research into their role as potential biomarkers for use in precision approaches to patient stratification for various diseases, including CVDs [32,33,34]. It is noteworthy that circulating miRNAs share optimal biochemical properties to be excellent biomarkers; specifically, they (1) are highly stable in an extracellular environment, (2) are easily detected in plasma samples and serum, (3) can be obtained by standard laboratory techniques, and (4) are protected from degradation due to their binding to proteins or their encapsulation in microvesicles [35].

## 3. Clinical Relevance of miRNA Networks in the Clinical Practice of CVDs

During the last two decades, miRNAs have shown tremendous potential to serve as diagnostic and prognostic biomarkers in the cardiovascular field. Their organ- and cell-specific regulation allow them to be studied in a variety of disease entities within the CVD spectrum. Despite several studies having shed light to the properties of miRNAs in the classification and risk stratification of patients with CVD [11,36], the validation of the reported results in large-scale clinical trials is still needed, particularly with regard to the potential clinical utility of miRNAs as therapeutic agents.

Circulating miRNAs are extremely stable in the circulation, and can be easily detected with high sensitivity and specificity using sequence-specific amplification [37]. Hence, they are considered to be able to serve as potential clinical biomarkers with great diagnostic power (non-inferior to the established protein-based biomarkers) and promising prognostic value in lipid metabolism disorders, atherosclerosis, and cardiomyopathy [15]. For example, several studies concerning miRNAs as potential biomarkers in atherosclerosis and in cardiovascular risk factors were published in the last few years; likewise, genome sequence variations, including SNPs, could act as biological markers for the response to certain drugs [38,39,40]. However, genomics alone cannot provide a complete monitoring of the patient’s phenotype. The miRNA expression reflects changes to distinct pathological conditions and drug treatments, indicating a complex interaction between genetic and environmental factors, and providing functional insights in disease prediction [41]. For example, a correlation between platelet-derived miRNAs’ expression levels with P2Y12 receptor inhibitor (clopidogrel and prasugrel) responsiveness has been proposed [42,43,44], and this association could be influenced by the interaction of CYP2C19 genotype polymorphisms [45]. Thus, innovative and more accurate biomarkers are still an unmet need in the monitoring of cardiovascular therapies, and could lead to personalized therapies. Novel candidate miRNAs have to be extensively validated based on -omics sciences along with improvements in risk prediction algorithms, and hold the potential to provide very accurate predictions at an early stage of CVDs. The clinical relevance of miRNAs within these aspects of CVDs will be described below.

## 4. Experimental Data Collection of miRNA CVD Biomarkers and Bioinformatic Analysis

MiRNAs associated with CVDs were retrieved from the mir2disease [46] and phenomir [47] databases using multiMiR [48]. Specifically, disease terms associated with CVDs were manually selected as input for performing queries to retrieve CVD-associated miRNAs (Table S1). Validated target genes (VTGs) of the CVD-associated miRNAs were retrieved from the mirtarbase [49] and mirecords [50] databases using multiMiR. For increased accuracy, miRNA-gene target interactions were further filtered to include only those that were experimentally validated by Reporter gene assays (e.g., Luciferase assay), Immunoblot/Western blot, and Real-time (RT) qPCR. Furthermore, information of CVD-associated drugs and SNPs that target and/or affect VTGs were retrieved from the Cardiovascular Disease Systems Pharmacology (CVDSP) database (https://old.tcmsp-e.com/cvdsp.php (accessed on 27 September 2021)) [51] and the study of Vizirianakis et al. [52], respectively. According to our analysis, miR-155-5p is by far the most prominent in the list of CVD-associated miRNAs. As shown in Table 1, according to mirtarbase and mirecords databases, miR-155-5p interacts with 241 different genes playing a central role in regulating genes contributing to human cardiac hypertrophy and hypertension, as retrieved by the mir2disease database. The list of top ten overrepresented miRNAs involved in CVDs is further supplemented by miR-21-5p, miR-145-5p, miR-34a-5p, miR-125b-5p, miR-29a-3p, miR-24-3p, miR-29b-3p, miR-200c-3p, and miR-17-5p which have far fewer gene targets than miR-155-5p. Most of them are related to cardiomyopathy, cardiac hypertrophy and stroke (Table 1).

**Figure 1:**
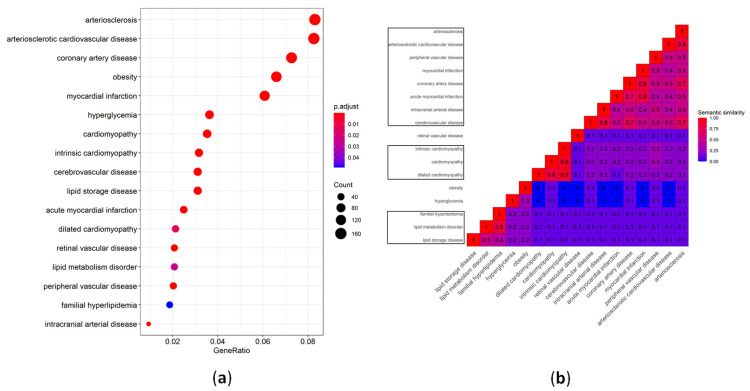
Selected CVD-associated DOSE terms that are enriched in the validated gene targets of CVD-associated miRNAs. (**a**) Dot plot of statistically significant, CVD-associated DOSE terms, displaying the gene ratio and statistical significance (*p*-value < 0.05, following Bonferroni adjustment). (**b**) Heat map displaying the semantic similarity between the CVD-associated DOSE terms. The DOSE term similarity measurements were performed using the method of Wang et al. [57] and clusterProfiler [55,56]. The DOSE terms were clustered using hierarchical clustering. Clusters with two or more terms are specified with black-colored borders. The heat map was created using ComplexHeatmap [58].

**Figure 2:**
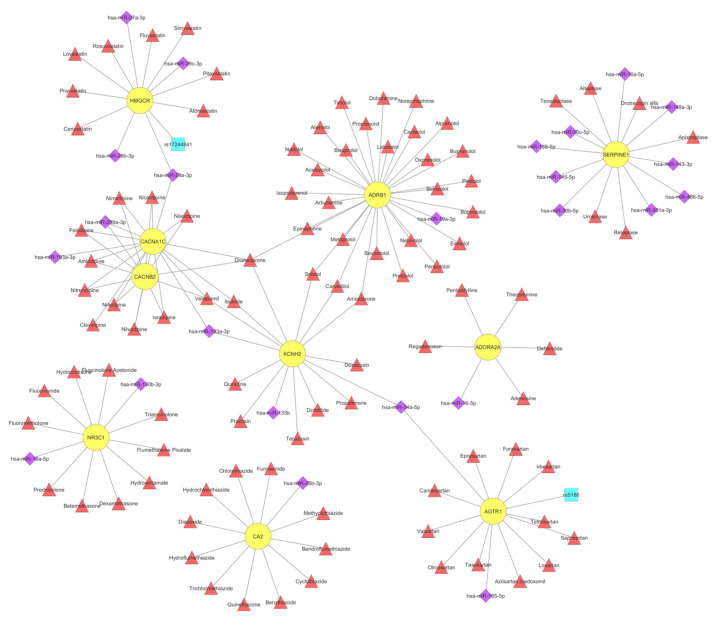
Network representation of ten gene-targets of CVD-associated miRNAs, which were also found to be targeted by most CVD-associated drugs from the CVDSP database [51]. The network displays 10 validated gene targets (circles in yellow), as well as 24 miRNAs (diamonds in purple), two SNPs (squares in turquoise) and 98 drugs (triangles in red) that are CVD-associated, and which target and/or affect them. The network was created using Cytoscape [59].

Over-representation analysis on VTGs was performed for disease ontology (DOSE) terms (Table S2) and Reactome pathways (Table S3), using DOSE [53], ReactomePA [54] and clusterProfiler [55,56]. The selected CVD-associated DOSE terms that were enriched in the VTGs of CVD-associated miRNAs are shown in Figure 1. In particular, as presented in Figure 1a, a larger number of genes (about 160) are associated with the terms “arteriosclerosis”, and “arteriosclerotic cardiovascular disease”. The top five categories identified are related to the terms “arteriosclerosis”, “arteriosclerotic cardiovascular disease”, “coronary artery disease”, “obesity”, and “myocardial infraction” (Figure 1a). The subsequently executed similarity analysis between the CVD-associated DOSE terms revealed three major clusters (Figure 1b) that will be further presented and discussed under the terms “atherosclerosis”, “cardiomyopathy”, and “lipid metabolism disorder”. As far as the pharmacogenomic association of the genes identified is concerned, the in silico analysis of the top ten most drug-targeted genes is shown in Figure 2.

## 5. Atherosclerosis

Accumulating evidence from numerous studies have demonstrated the significant role of miRNAs in the development of atherosclerosis, controlling the molecular and cellular mechanism of onset and progression of atherosclerosis by acting in different vascular cells. The major cell categories affected by miRNAs are endothelial cells (EC), inflammatory cells, mainly macrophages and monocytes and vascular smooth muscle cells (VSMC). Although the vast majority of miRNAs are expressed in all atherosclerosis-associated cell types (ECs, macrophages and VSMCs), a small group of them were expressed in a cell-type specific manner [60]. In addition, their expression is highly regulated at a post-transcriptional level as evidenced by the inconsistency between pri-miRNA and mature miRNA expression [60] and by the fact that many pri-miRNAs fail to produce mature miRNAs due to their processing [61].

**Figure 3:**
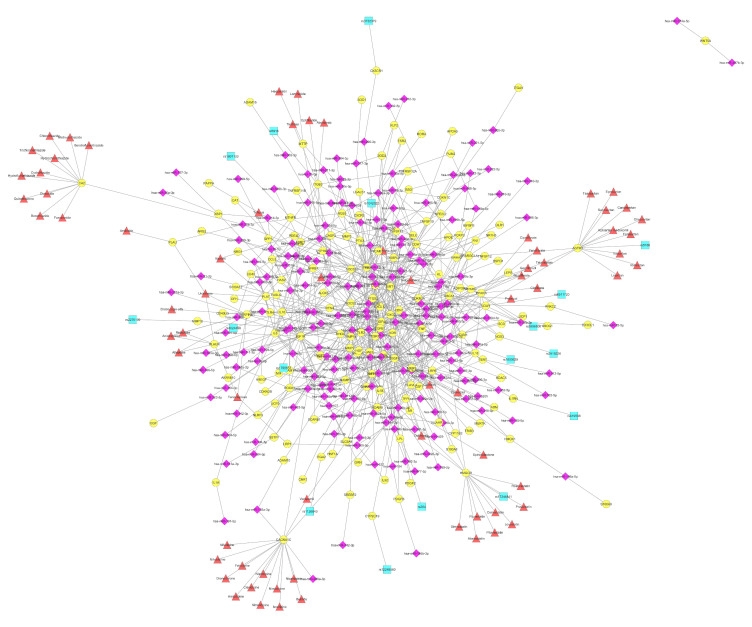
Network of VTGs enriched within the DOSE term arteriosclerosis. The network displays 160 arteriosclerosis-associated VTGs (circles in yellow), as well as 196 miRNAs (diamonds in purple), 17 SNPs (squares in turquoise) and 70 drugs (triangles in red) that are CVD-associated, and target and/or affect them. The network was created using Cytoscape [59].

In our analysis, under the term “atherosclerosis”, many different terms with high similarity have been grouped and discussed together. These terms are: “arteriosclerotic cardiovascular disease”, “arteriosclerosis”, “retinal vascular disease”, “coronary artery disease”, “hyperglycemia”, “myocardial infarction”, “obesity”, “peripheral vascular disease”, “cerebrovascular disease”, “acute myocardial infarction”, “cardiomyopathy”, and “intracranial arterial disease” (Figure 1b). As shown in Figure 3, the complicated network describing the interactions of VTGs with CVD-associated miRNAs consists of 724 connections. More than 190 miRNAs have been found to be involved in the regulation of 160 different genes in all phases of atherosclerosis (Figure 3). In particular, the average number of connections found between a VTG and miRNAs is 4.5, while the vast majority of them (114 out of 160; 71.25%) had less than five connections, and 17 out of 160 (10.63%) interacted with more than 10 candidate miRNAs. Of the 196 different miRNAs associated with atherosclerosis, the ten with the most gene interactions are presented in Table 2. Of these, the most abundant, miR-146a-5p, as well as miR-143-3p, miR-221-3p, miR-126-3p, and miR-138-5p are absent from the list of miRNAs with the most gene interactions implicated in CVDs (Table 1). MiR-146a-5p was found to target 18 genes—namely *CCL5*, *CD40LG*, *CFH*, *CXCL12*, *ELAVL1*, *ICAM1*, *IL6*, *MIF*, *NFKB1*, *PLAUR*, *PTGS2*, *RHOA*, *ROCK1*, *S100A12*, *SPP1*, *TGFB1*, *TLR2*, and *TLR4*—associated with the development of atherosclerosis. The role of miR-146a in atherosclerosis has been described; its increased expression has been correlated with an increased risk of atherosclerosis in patients with CAD [62]. Moreover, in patients with hypothyroidism, it counts as a potential predictor for atherosclerosis [63].

The maximum number of interactions with different miRNAs was 42, and was observed for the vascular endothelial growth factor (*VEGFA*) gene. VEGFA is the most functionally important member of the Vegf family [64], and was initially identified as an inducer of vascular permeability (vascular permeability factor, VPF). VEGFA is a proangiogenic factor [65] that—through binding to its receptor, mainly Vegfr-2/Flk1/Kdr—stimulates haematopoietic cell proliferation, survival, differentiation and migration [66]. Indeed, numerous miRNAs—including miR-15a, miR-200b, miR-20b, miR-1, miR-206, and miR-93—have been discussed extensively in the literature to target *VEGFA*, and have been validated with multiple methods (Table S4). Additionally, 17 different SNPs have been identified in an equal number of genes (Table S1). Regarding the therapeutic drugs, 24 genes are serving as targets for 70 different compounds (Table S1). The largest group of drugs (13), mainly containing calcium channel blockers, target the *CACNA1C* gene. This gene encodes for the alpha-1 subunit of a voltage-dependent calcium channel that controls the flow of calcium ions into cardiomyocytes. In parallel, *CACNA1C* was found to interact with miR-103a-3p, miR-133a-3p, miR-208a-3p, and miR-29a-3p (Figure 3). *CACNA1C* has been validated as a target of miR-103a-3p, one of the most important miRNAs associated with recurrent venous thromboembolism [67]. Moreover, the overexpression of miR-29a-3p was associated with the reduced expression of *CACNA1C* in patients with atrial fibrillation, enhancing its potential therapeutic role in its treatment [68].

According to our analysis, the four genes—*AGTR1*, *HMGCR*, *ITGB3,* and *PPARG*—shown to be influenced by specific miRNAs, or targeted by drugs and SNPs in their sequence are also associated with atherosclerosis (Figure 3). Notably based on this fact, rs5186 (Chromosome 3:148742201) in the *AGTR1* gene, rs17244841 (Chromosome 5:75347030) in the *HMGCR* gene, rs5918 (Chromosome 17:47283364) in the *ITGB3* gene, and rs3856806 (Chromosome 3:12434058) in the *PPARG* gene were also included in our clinical trial GESS (NCT03150680), in order to assess any potential predictive value with the SYNTAX score; this outcome, if exists upon the completion of the study, it will be of great interest in the clinical setting of CVDs.

Angiotensin II receptor type 1 (*AGTR1*, *AT1R*) encodes for multiple transcript variants of the type 1 receptor of angiotensin II, which is an important effector controlling blood pressure and volume in the cardiovascular system. *AGTR1* expression levels are largely regulated by post-transcriptional mechanisms [69]. MiR-155 was found overexpressed in human umbilical vein endothelial cells (HUVECs) and vascular smooth muscle cells (VSMCs) and by using Western blot, it was confirmed that *AT1R* is a target of miR-155 in HUVECs [70] (Table S4). Zheng et al demonstrated that overexpression of miR-155 in rat’s aortic adventitial fibroblasts significantly reduced AT1R protein expression despite that mRNA levels remained unaffected [71]. Luciferase reporter assays further confirmed that miR-155 interacts directly with the AT1R 3′-UTR and translationally represses the expression of this protein in vivo [71]. Of interest, the SNP +1166A/C or A1166C (rs5186), which is located in the 3’ UTR of *AT1R* gene is recognized by miR-155 [72] and results in altered AT1R protein expression in a cohort of hypertensive patients [73] or in patients with advanced carotid atherosclerosis [74]. The drugs related to *AGTR1* gene, such as candesartan, eprosartan and olmesartan, are angiotensin II receptor blockers (ARBs) that are widely used to treat high blood pressure (Figure 2 and Figure 3).

3-hydroxy-3-methylglutaryl coenzyme A reductase (HMGCR) is an atheroprotective enzyme that limits cholesterol biosynthesis, preventing CVD and other atherosclerotic diseases [75]. MiR-29 directly targets *HMGCR* as evidenced by decreased levels of luciferase activity in the presence of a miR-29a mimic [76] (Table S4). Using Dicer1-knockout mice, Liu et al found that miR-29a/b/c significantly suppressed *HMGCR* expression (by targeting the *HMGCR* mRNA 3′-UTR) revealing the importance of miR-29 in regulating hepatic cholesterol homeostasis and as a potential therapeutic target [77]. In addition, miR-27 was found capable of significantly suppressing cholesterol biosynthesis by regulating *HMGCR* gene [78]. The genetic polymorphism rs17244841 in *HMGCR* gene, although relatively rare, caused a significant reduction in total cholesterol and LDL-c levels in heterozygous CAD patients treated with statins [79].

As mentioned above, one of the three vital cell categories in atherosclerotic plaque formation is smooth muscle cells (SMCs). Particularly in rupture caps, fewer SMCs are present, indicating an unstable plaque prone to rupture [80,81]. As was demonstrated by Misra et al., the reduction of integrin β3 (Itgb3, GPIIIa) levels in SMCs induces TLR4 expression, consequently enhancing Cd36 levels [82]. Moreover, the Itgb3 mutant phenotype resulted in the recruitment of multiple pre-existing SMCs into plaques, while a medium conditioned with Itgb3(−/−) macrophages increased SMC migration and proliferation [82]. Altogether, the important contribution of Itgb3 in atherogenesis has been highlighted. Several miRNAs have been found in our analysis to interact with *ITGB3*, namely let-7a-5p, let-7c-5p, miR-17-3p, miR-30a-5p, miR-30c-5p, and miR-98-5p (Table S4). Moreover, the rs5918 (PlA1/A2) polymorphism in the integrin β3 gene is a well-established risk factor for acute coronary thrombosis and premature myocardial infarction [83,84]. Carriers of the Leu33Pro polymorphism have been linked to an increased risk of atherosclerotic plaque rupture in an ARIC study in a Caucasian population [85]. Three therapeutic choices were found to target GPIIIa in order to prevent platelet aggregation (Figure 3). Abciximab is a monoclonal anti-glycoprotein IIb/IIIa receptor antibody, while eptifibatide and tirofiban are a peptide-based antagonist and a non-peptide reversible antagonist of the platelet glycoprotein IIb/IIIa receptor, respectively.

The peroxisome proliferator activated receptor gamma (*PPARγ*) gene is located in chromosome 3 (3p25), and encodes for a protein that is a member of the PPAR family. The three members of the PPARs—namely PPARα, PPARγ and PPARβ/δ—are ligand-activated transcription factors of nuclear hormone receptor that play a vital role in the regulation of energy homeostasis and metabolism, but each of them performs unique functions in these processes and displays tissue-specific expression patterns [86,87,88]. PPARγ is an essential regulator of adipocyte differentiation and glucose homeostasis, and is highly expressed—in addition to adipocytes—in VSMCs, in ECs, and in macrophages [89]. The significant contribution of PPARγ in the limitation of the progression of atherosclerosis through multiple mechanisms is well studied and reviewed elsewhere [89]. Our in silico analysis revealed seven miRNAs that interact with PPARγ (Figure 3, Table S4). MiR-130 strongly suppresses *PPARγ* expression, resulting in the inhibition of adipocyte differentiation. Their interaction was both in the PPARγ mRNA-coding region and in 3′ UTR, as shown by Lee et al [90]. Interestingly, in the same study, obese women showed significantly lower miR-130 levels and increased PPARγ mRNA levels in comparison with lean women [90]. Similarly, an upregulation of miR-27b, miR-130b and miR-138 in obese colorectal cancer patients was observed, while PPARγ was downregulated [91]. Likewise, miR-130 and miR-27a/b are known adipogenic inhibitors that directly target the major regulators of adipogenesis, *PPARγ* and *C/EBPα* by suppressing their expression [92]. MiR-20 has been shown to promote osteogenic differentiation in mesenchymal stem cells (MSCs), and it was confirmed using different assays that *PPARγ*, *Bambi* (BMP and activin membrane-bound inhibitor) and *Crim1* (Cysteine-rich motor neuron 1) are direct targets of miR-20a [93]. Regarding the role of the *PPARγ* polymorphism rs3856806 at the onset of atherosclerosis, the results are contradictory. In a study with 787 Iranian individuals with CAD and dyslipidemia, it was shown that C1431T polymorphism was significantly associated with fasted serum lipid levels [94], while in a study with 2102 children from Greece, the CT+TT genotypes of C1431T polymorphism were associated with increased adiposity [95]. In addition, a meta-analysis study of eight studies confirmed the association of C161T polymorphism with an increased risk of [96]. On the contrary, the meta-analysis by Wang et al involving 29 studies failed to support a significant correlation of rs3856806 with the risk of atherosclerotic disease in general, except for the Caucasian and myocardial infarction subgroups [97]. Considering the current literature, further investigation is needed to shed light on the importance of rs3856806 in the development of atherosclerosis.

## 6. Cardiomyopathy

**Figure 4:**
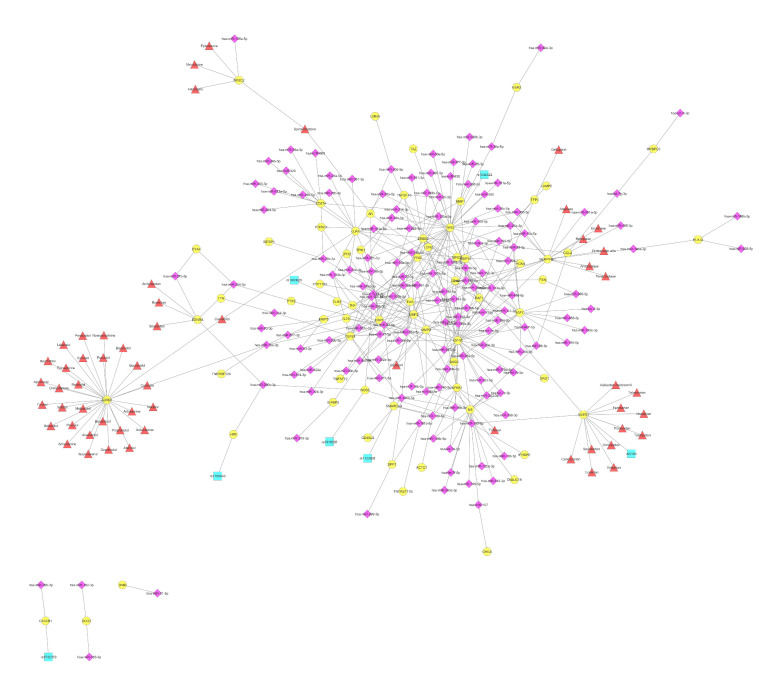
Network of VTGs enriched within the DOSE term cardiomyopathy. The network displays 67 cardiomyopathy-associated VTGs (circles in yellow), as well as 145 miRNAs (diamonds in purple), seven SNPs (squares in turquoise) and 56 drugs (triangles in red) that are associated with CVD, and which target and/or affect it. The network was created using Cytoscape [59].

Cardiomyopathy refers to a collection of disorders of the heart muscle that lead to a reduced ability to pump blood efficiently. According to our in silico analysis, in this category the terms “cardiomyopathy”, “intrinsic cardiomyopathy”, and “dilated cardiomyopathy” are grouped together (Figure 1b). In general, 340 interactions between 67 genes and 145 unique miRNAs were identified, which is substantially fewer than those described for atherosclerosis, although the number of miRNAs remained roughly equal (Figure 4). Moreover, the average number of connections found between a VTG and miRNAs was 5.1, while most of the genes (47 out of 67; 70.15%) had less than five connections, and 10 out of 67 (14.93%) interacted with more than 10 candidate miRNAs. As shown in Table 2, the miRNAs that target most of the genes associated with cardiomyopathy are miR-21-5p, miR-24-3p, miR-145-5p, miR-138-5p, miR-143-3p, miR-17-5p, miR-155-5p, miR-125b-5p, miR-146a-5p, and miR-133b and six of them are also in the list of the most abundant in CVDs in general (Table 1). Of the 145 different miRNAs implicated in cardiomyopathy, miR-21-5p was found to target 10 genes, namely *ERBB2*, *IGF1R*, *IL1B*, *MMP2*, *MMP9*, *PTX3*, *SMARCA4*, *TGFB1*, *TLR3,* and *TPM1* (Figure 4). MiR-21 has gained great attention because of its involvement in many biological processes and clinical conditions, and has also received increased attention due to its involvement in many biological processes, primarily in CVD. The increased expression of miR-21 was observed in the left ventricles of patients with dilated cardiomyopathy (DCM), but not in hypertrophic cardiomyopathy (HCM) [98]. In addition, there was no significant difference in the circulating miR-21 levels in patients with ischemic cardiomyopathy and DCM, but they were found to be significantly increased compared with the control group [99], suggesting their value as a biomarker for heart failure.

The Beta-1 adrenergic receptor (*ADRB1*) gene is by far the most drug-targeted gene, with 29 different compounds from the total 56 different drugs that have been identified to interact with genes associated with cardiomyopathies (Figure 4). Ser49Gly genetic polymorphism in the *ADRB1* gene was associated with a low mean resting heart rate in a cohort study of >1000 individuals from China and Japan [100], while in another study Ser49Gly polymorphism was associated with reduced mortality risk in patients with congestive heart failure, probably through myocardial protection [101]. As shown in Figure 2, beta-adrenergic receptors are principal targets for beta-blockers, such as bisoprolol, metoprolol, propranolol and atenolol to treat angina, hypertension and heart failure.

Tumor protein P53 (*TP53*), which encodes for a tumor suppressor protein which is responsible for the regulation of the tumor-free survival of organisms, is regulated by 34 miRs (Table S4), which represent the highest number of miRNA-VTGs interactions (Figure 4). In cardiomyocytes, Tp53 plays a critical role in the regulation of the cardiac transcriptome [102], while elevated Tp53 levels have been associated with cardiac hypertrophy and remodeling [103]. Among the identified miRNAs, miR-34s has been extensively studied and recognized as the most prevalent miRNA induced by p53, not only in cancer where it suppresses tumor growth and metastasis, but also in non-cancerous diseases such as brain disorders, metabolic and cardiovascular diseases [104]. One of the most important miRNAs, miR-125b, which was the first discovered miRNA [105], has been also found to interact with *TP53*. In the latest study of Chen et al, cardiac-specific miR-125b-1 knockout mice were generated that displayed dysregulated fatty acid metabolism, leading to perinatal death and cardiac hypertrophy [106], a condition that eventually leads to cardiomyopathy and heart failure. 

*AGTR1* is the only gene out of the 67 cardiomyopathy-associated genes that revealed a connection with at least one miRNA, drug, and SNP. In particular, in full accordance with what is described in the atherosclerosis section, *AGTR1* is a target for miR-155-5p, miR-34a-5p, and 11 drugs acting as angiotensin II receptor type 1 (AT_1_) antagonists, with its most widely studied polymorphism, rs5186, to be also associated in the connection map (Figure 4).

## 7. Lipid Metabolism Disorder

**Figure 5:**
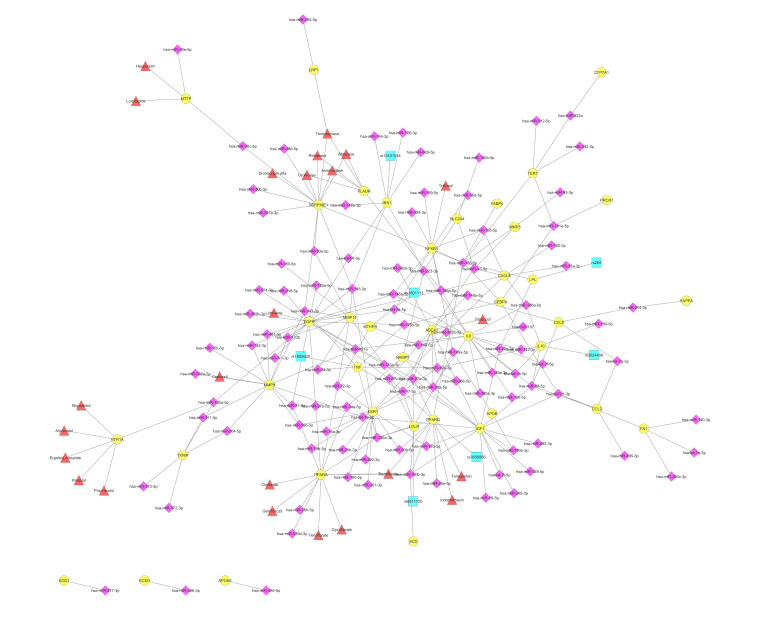
Network of the VTGs enriched within the DOSE term lipid metabolism disorder. The network displays 40 lipid metabolism disorder-associated VTGs (circles in yellow), as well as 109 miRNAs (diamonds in purple), seven SNPs (squares in turquoise) and 24 drugs (triangles in red) that are associated with CVDs, and target and/or affect them. The network was created using Cytoscape [59].

Dyslipidemia is one of the most important risk factors in the pathophysiological basis of CVDs and the development of atherosclerosis. Blood lipids are a complex environment that includes cholesterol, low-density lipoprotein (LDL), high-density lipoprotein (HDL), lipoprotein A (Lp(a)), triglycerides and fatty acids. The most important lipid disorders affecting CAD occurrence risk are those related to an increased serum LDL level or a reduced HDL level. Despite the extensive research in the field and the development of lipid-lowering therapeutics such as statins that inhibit cholesterol synthesis, alternative treatment options and therefore novel pharmacological targets need to be discovered. According to our analysis under the term “lipid metabolism disorder”, the terms “lipid storage disease”, “lipid metabolism disorder” and “familial hyperlipidemia” are clustered (Figure 1b). The network presenting the correlations between VTGs associated with lipid metabolism disorder and miRNAs consists of 215 interactions, as shown in Figure 5. In general, 40 genes were recognized to interact with 109 different miRNAs, while 24 therapeutics target 11 of them. The most common miRNA in the lipid metabolism network is hsa-miR-138-5p, which is found to target eight different genes, namely *CEBPA*, *FABP4*, *LPL*, *MMP3*, *NFKB1*, *SERPINE1*, *PPARG* and *TERT*. In addition to miR-138-5p, the list of miRNAs with the most interactions with genes involved in lipid metabolism disorders is complete with miR-146a-5p, miR-26a-5p, miR-27a-3p, miR-145-5p, miR-130a-3p, miR-98-5p, miR-130b-3p, miR-223-3p, and miR-1-3p (Table 2). Except of miR-145-5p, all other miRNAs are not listed in Table 1 with most abundant miRNAs being involved in CVDs (Table 1). The most interactions were observed between epidermal growth factor receptor (*EGFR*) and 20 miRNAs (Table S4). Among them, miR-125a-5p and miR-7 were recently verified, by Western blot analysis, to target *EGFR* and thereby stimulate the growth, migration and invasion of VSMCs [107]. On the contrary, the study of Prasad et al, on the explanted hearts of transplant recipients with a diagnosis of DCM showed that the overexpression of miR-7 significantly reduces the expression of *ERBB2* but not *EGFR*, in contrast to cancer cells [108,109]. Most of the myocardial energetic requirements in adults are derived from the oxidation of free fatty acids. In CVDs, the myocardial energy metabolism is disrupted, and a lack of energy or excessive fat accumulation is observed [110]. As was mentioned above, PPARγ is a major regulator of adipogenesis. According to our in silico analysis, *PPARγ* is the only VTG associated with lipid metabolism disorder that revealed interactions with miRNAs and is also a potential therapeutic target.

Following this, the existing knowledge concerning miRNA biomarkers within each cluster of the three CVD terms—namely (a) atherosclerosis and coronary disease, (b) cardiomyopathy and heart failure, and (c) lipid metabolism disorders—will be discussed separately. This direction could advance pharmacogenomics-guided decisions and practical clinical utility by easily facilitating correlations between common CVD pathophysiological features, drug-gene targets, SNPs, and miRNA-genomic networks in different CVD clinical conditions.

## 8. The Clinical Implementation of the Biomarker miRNA Networks in the CVD Cluster of Atherosclerosis and Coronary Artery Disease

Atherosclerosis, the main cause of CAD, is a progressive inflammatory disease of the arterial wall, leading to endothelial dysfunction and apoptosis, lipid accumulation, and the subsequent formation of atheromatous plaques [111,112]. The occlusion of a coronary artery as a result of an atherosclerotic process can lead to acute MI, and to cell death due to the prolonged deprivation of the oxygen supply.

Despite miRNAs’ infancy in the research community, their ability to regulate protein expression has led to their implementation as a means to achieve earlier diagnoses and better therapeutics to combat the initiation, progression, and complications of atherosclerosis [113]. To date, the accumulated evidence has associated specific miRNA expression patterns with the development of atherosclerosis and CAD, which are two closely related conditions. This occurs due to miRNA’s participation in relevant molecular pathways, including endothelial dysfunction, cellular adhesion, proliferation, lipid uptake and efflux, the generation of inflammatory mediators, platelet and VSMC activation, and plaque formation [37,38]. A recent systematic review supported the hypothesis that some miRNAs may be involved in atherosclerotic expression in specific territories (carotid, inferior limbs/arteriosclerosis obliterans, renal artery), while others may be involved in the common mechanisms of atherosclerosis [114]. Several miRNAs can be delivered by apoptotic bodies to the atherosclerotic lesions [115]; therefore, the reduction of circulating miRNAs in patients with atherosclerosis/CAD might be attributed to an uptake of circulating miRNAs into atherosclerotic lesions [116,117].

Plenty of studies on patients with CAD, have demonstrated the downregulated expression of endothelial cell-enriched miRNAs (miR-126, miR-17, and miR-92a), and VSMC-enriched miRNAs (miR-145) [118,119]. In contrast, cardiomyocyte-enriched miRNAs (miR-133 and miR-208a) were upregulated [118]. The expression of pro-inflammatory cell-enriched miRNAs (miR-155) was reported to be either increased or reduced in patients with atherosclerosis [118,120].

Other research groups showed that patients with CAD had higher plasma levels of miR-133a, miR-150, miR-34a, miR-21, miR-30a, miR-146a, miR-146b, miR-106b, miR-206, miR-574-5p, miR-221, and miR-222 when compared to controls, while the ratio of miR-135a to miR-147 concentration was 19-fold higher in patients with CAD in comparison with healthy individuals [121,122,123,124]. On the contrary, patients with CAD had lower levels of miR-126-5p, miR-17-92, miR-31, and miR-720 when compared to healthy patients [125,126,127]. 

Nowadays, miRNA diagnostic panels have been developed to enhance discriminatory power for the detection of patients with atherosclerosis and CAD. In particular, miR-765, miR-149, and miR-424 were useful to discriminate both patients with stable and unstable CAD from controls [128]. Other combined panels comprise of: i. miR-24, miR-33, miR-103a, and miR-122 [129], ii. miR-155, -145, and let-7c [130] and iii. miR-132, miR-150, and miR-186 [131], which can provide a high diagnostic accuracy on CAD even in higher levels than cardiac TroponinI (cTnI) alone.

Additionally, miRNAs can also serve as the biomarkers which are required to differentiate Type I MI–due to atherosclerotic-plaque rupture (upregulated miR-1, miR-133a, miR-133b, and miR-499-5p, and downregulated miR-122 and miR-375) from Type II–MI due to supply/demand mismatch [132,133,134]. With regard to the subtype of MI, Ward et al. reported that miR-25-3p, miR-221-3p, and miR-374b-5p were strongly correlated with ST-Elevation Myocardial Infarction (STEMI), while miRs-221-3p and -483-5p were highly associated with non-ST-Elevation Myocardial Infarction (NSTEMI) [135]. The upregulation of a few other miRNAs (miR-134, miR-198, and miR-370) was uniquely linked to unstable angina (UA) compared to stable CAD, and can facilitate the diagnosis of UA, which can be elusive in some patients with normal troponin values [136].

Furthermore, a combined model of miR-483-5p and miR-451a can differentiate plaque rupture [125], while circulating miR-133a, miR-208a, miR-155, miR-145, miR-214, and miR-223 were strongly correlated with the severity of CAD [137,138,139,140]. Other studies suggest that circulating miRNAs (miR-19a, miR-208a, miR-133, and miR-499) might be detectable even earlier than the traditional prognostic biomarkers (cTnI, creatine kinase-MB (CK-MB), and brain natriuretic peptide (BNP)) [118,141]. Thus, in the near future, an miRNA panel, probably in conjunction with traditional biomarkers, might have a role to play in the emergency department, leading to the diagnosis of acute coronary syndrome with great sensitivity and accuracy.

The current miRNA therapeutics for atherosclerosis treatment is focused on the systemic administration of either anti-miRNAs (antagomirs) or synthetic miRNA mimics (miRNA mimetics), by using viral and non-viral delivery platforms as vehicles [142]. The delivery of miR-126 by apoptotic bodies was shown to counteract atherosclerosis, inducing vascular protection and facilitating the “fine-tuning” of atherosclerotic disease [143]. Other well-performed studies have demonstrated the cardioprotective potential of intercellular communication mechanisms by miRNA-containing extracellular vesicles [144]. Therapeutic miR-210 delivery has also been linked with the decreasing of the infarct size, as well as the improvement of angiogenesis and cardiac function [145].

Moreover, miRNAs may pave the way for personalized therapy in patients with atherosclerosis/CAD. They could potentially be used to identify patients who could benefit from antiplatelet treatment. This was evaluated in studies by measuring plasma miRNA’s response to antiplatelet treatment and platelet activation markers at different moments in the course of the treatment. Several platelet-related miRNAs (miR-223, miR-191, miR-126, and miR-150) were decreased as a result of platelet inhibition in the plasma (via P2Y12 expression pathway modulation) [35,43]. Hence, circulating miRNAs can provide tailored, effective antiplatelet therapy by monitoring its efficiency and detecting non-respondent patients.

## 9. The Clinical Implementation of the Biomarker miRNA Networks in the CVD Cluster of Cardiomyopathy and Heart Failure

Despite BNP and N-terminal (NT)-pro hormone BNP (NT-proBNP) being well-established gold-standard biomarkers for the evaluation of HF and cardiomyopathy progress, miRNAs seem to be able to play a role in the management of HF that frequently occurs in patients with cardiomyopathies [36,116,146]. Recently emerging evidence suggested, for the first time, that miRNA expression patterns (miR-1, miR-16, miR-21, miR-26a, miR-133, miR-146a, and miR-192) could differ in various forms of cardiomyopathy (hypertrophic, dilated, ischemic, diabetic, Tako-tsubo, peripartum, and pressure overload), especially when compared to that of normal hearts, thereby constituting biomarkers of high diagnostic and discriminatory value [147,148,149,150,151,152,153,154,155,156,157,158,159]. Moreover, upregulated miR-192 expression was independently associated with decreased survival rates in patients with ischemic cardiomyopathy but not in patients with non-ischemic cardiomyopathy [160], while left ventricular assist device treatment has succeeded in normalizing abnormal miRNAs expression in ischemic cardiomyopathy [161].

In particular, DCM is a complex disease with a common phenotype but heterogeneous pathological mechanisms, and early etiological diagnosis and risk stratification seems to be crucial for the clinical course of the disease. Despite the state-of-the-art imaging technology and genetic tests provided, novel non-invasive markers are still needed to guide the clinical decision-making [162]. To that end, miRNAs are emerging as a promising tool for the clinical management of DCM because it is intimately associated with an altered intracellular and circulating miRNA profile. MiR-21 was upregulated, and miR-29a, miR-29b, miR-29c, miR-133a, and miR-133b were downregulated in the apex, left, and right ventricles of patients with DCM compared with normal cardiac tissues [163]. MiR-1-3p and miR-27a were differentially expressed among the left ventricles of HCM and DCM patients with an optimal discrimination performance, with an area under the receiver operating characteristic curve (AUC) > 0.850 [98]. With regard to the DCM prognosis, patients with end-stage DCM showed a consistent miRNA pattern, with a downregulation of miR-7 and miR-378, and an upregulation of miR-214, miR-181b, and miR-423-5p [164]. In DCM, some myocardial miRNAs were found to predict the time-dependent reverse-remodeling response to β-blocker treatment [165]. Finally, anti-miR-34a therapy was shown to be more effective in females with moderate DCM than in males [166].

Mounting evidence suggests that miRNAs are involved in the development and progression of HF in general. Findings from different studies in patients with HF revealed an upregulation of some miRNAs (miR-21, miR-23a, miR-125b, miR-195, miR-199A, miR-214, and miR-342) and a downregulation of others (miR-1, miR-7, miR-29b, miR-30, miR-133, miR-150, and miR-378) [167]. Analogous to atherosclerosis, the combination of two or more miRNAs as a defined set (an miRNA signature) enhances the discriminatory power in HF as well. A study on HF with a reduced ejection fraction (HFrEF) identified eight miRNAs (miR-520d-5p, miR-558, miR-122, miR-200b, miR-622, miR-519e, miR-1231, and miR-1228) that reliably predicted the diagnosis of HFrEF (AUC = 0.81), and demonstrated a potential superiority of miRNA signatures over the quantification of single miRNA for diagnostic purposes [167,168].

As for HF with a preserved ejection fraction (HFpEF), three recent clinical studies assessed the capacity of circulating miRNAs to predict the differentiated diagnosis of HFPEF vs HFREF [169,170,171]. However, none of the identified miRNAs were reported more than once, and no validating results could be presented so far [169]. This emphasizes the need for the further development of larger clinical trials evaluating miRNAs in this disease entity. In particular, the exploration of signaling pathways and validation analyses in further clinical cohorts are of utmost importance [172].

## 10. The Clinical Implementation of the Biomarker miRNA Networks in the CVD Cluster of Lipid Metabolism Disorders

MiRNAs were reported as critical regulators of cholesterol metabolism, and genetic variations in miRNA loci were associated with alterations in human cholesterol and triglyceride levels. It is noteworthy that miRNAs have a two-way relationship with HDL cholesterol because they regulate several pathways of its metabolism, while at the same time HDL molecules provide a vehicle for circulating miRNAs [173]. Recent studies underline the potential utility of miRNA mimics and inhibitors in the treatment of lipid metabolism disorders. The association of miR-33 and miR-122 with the control of HDL cholesterol metabolism and other cellular functions associated with CVD has been demonstrated well in many studies [174]. The experimental inhibition of miR-33 and miR-122 in dyslipidemic mice by antisense oligonucleotides resulted in a marked increase in HDL levels and a significant decrease in VLDL levels within three months by amplifying cholesterol export and fatty acid β-oxidation [175,176]. However, some studies of the genetic ablation or chronic antagonism of these miRNAs in conjunction with the Western diet have reported adverse effects, including obesity, increased circulating triglyceride, hepatic steatosis, and hepatocellular carcinoma [177]. Further studies are also warranted to determine whether the silencing of miR-128-1 and miR-148a could alleviate dyslipidemia, obesity and insulin resistance [174,178].

On the other hand, high levels of miR-146a and miR-30c seem to have a positive effect on lipid metabolism by down-regulating lipid synthesis and lipoprotein secretion. Thus, their overexpression might be an attractive approach for the treatment of homozygous hypercholesterolemia by attenuating the intracellular lipid accumulation [179,180]. However, the inability to predict the exact mechanisms by which miRNAs exert their phenotypic effects raises some concerns about the clinical implementation of miRNA-based anti-hyperlipidemic treatments. Therefore, further experiments are necessary to elucidate the effects of individual miRNA targets on specific phenotypes, thereby facilitating the outcome evaluation of miRNA-based clinical trials for dyslipidemia [174].

## 11. Discussion

The practical clinical utility needed upon the translation of molecular information is stressfully evident upon the analysis of the application of pharmacogenomics (PGx) knowledge and biomarkers in the development of innovative therapeutics for CVDs. Our methodological bioinformatic approach and the data derived from this analysis contribute to uncover pharmacogenomics-guided therapeutic decisions in cardiovascular precision medicine, and to dissect miRNA-gene networks by mapping clinical utility roads for biomarkers’ implementation in diagnosis, treatment, and research. The latter is crucial because the new FDA drug approval with PGx labeling within the period of 2000–2020 in the era of cardiology was only 4.5%, compared to those for other therapeutic areas, i.e., oncology, with 49.4%; neurology, with 9.0%; infectious diseases, with 7.9%; psychiatry, with 5.6%; and inborn errors of metabolism, with 5.1% [181]. A similar demand is observed upon applying methodologies which are capable of exploiting genomic biomarkers in designed and executed clinical trials in the era of CVDs [182]. Furthermore, it was emphatically shown that the successful broad implementation of the concepts of precision cardiovascular medicine in the clinical setting presupposes that the pleiotropic molecular associations underlying biological pathways must be efficiently solved and mapped before the application of biomarkers for specific pathophysiological conditions [9]. Moreover, this direction must be seen in parallel with the practice-changing breakthroughs which have already taken place in cardiovascular medicine within the last couple of decades, but also with those advances envisioning machine learning technology, wearable devices and e-mobile data that pave the way for the achievement of the goal of the individualization of precision medicine [183]. To this end, the data presented in this article attempt to uncover clinical knowledge of practical utility in CVDs through the application of a pipeline bioinformatic analysis to map clinically-relevant and druggable miRNA-gene networks, along with the genomic signatures resulting from SNPs.

It is known that microRNAs affect cardiac cell development and heart-tissue homeostasis [184]. On the other hand, miRNA biogenesis presents dynamic nature in terms of the molecular pathways involved and the gene-target mechanisms underlying the miRNA-mediated regulation. Moreover, the maturation, secretion, transfer, uptake, and function of extracellular miRNAs in disease pathophysiology, such as CVD conditions, is still elusive in research and the clinical practice [185]. Differences in miRNA biology may also count in the case of acute and chronic pathophysiological CVD conditions, sex differences, and existing comorbidities [186]. This existing knowledge gap in miRNA biology clearly hampers the effective clinical experimentation and subsequent exploitation of miRNAs as reliable and concise biomarkers in the clinical setting of CVDs. In addition, the synthesis of miRNAs from tissues other than the cardiovascular system along with their subsequent release into plasma as extracellular vehicles (e.g., exosomes), adds further barriers towards the exploitation of miRNAs as circulating biomarkers in CVDs. To overcome such issues and rigorously validate reliable miRNA biomarkers for CVDs, it is urgent need the development of novel experimental in silico, mathematical and artificial intelligence methodologies to be applied in clinical trial design, research, and healthcare. To this end, it is interesting to note the recent development of an algorithm capable to trace microRNA organismal origin from sequencing data [187]. Also, the creation and application of physiologically-based pharmacokinetic (PBPK) models for the development of innovative genomics and nanotechnology therapeutics, along with their integration with bioinformatics tools, present promise for the successful clinical exploitation of the molecular knowledge, including miRNAs, in the years to come [3].

Many parameters were reported to alter the expression of miRNAs, and should therefore be considered before the interpretation of the results. First of all, it is argued that the quantification of miRNAs might be affected by the baseline characteristics of concomitant medication, such as statin, heparin, aspirin, and angiotensin-converting enzyme (ACE) inhibitor prescriptions [43,188]. Specifically, statins seem to decrease circulating miR-122 levels, antiplatelet drugs seem to alleviate the amount of freely circulating thrombocyte-derived miRNAs, and heparin seems to influence the polymerase chain reaction during the quantification process [189]. Moreover, renal function may significantly modify the circulating levels of miRNAs [190,191].

The major limitations of the use of miRNAs as novel biomarkers in everyday clinical practice are their laborious isolation, complex detection procedures, questionable cost-effectiveness, and non-standardized normalization methods [192], while there is still no consensus as to whether plasma or serum is a more reliable substrate for their measurement [116]. The integration of the use of miRNAs in everyday clinical practice will require the development of accurate, rapid, and inexpensive techniques. Furthermore, a substantial number of studies were either based on animal models or conducted on a small sample of human participants. Many of those studies have used different protocols, and often yielded contradictory conclusions about specific miRNA expression. Hence, larger-scale trials are warranted to assess the potential utility of miRNAs either as biomarkers or therapeutic molecules. Although most of the miRNAs studied are rarely superior to traditional biomarkers in terms of diagnostic accuracy, they can be used effectively as supplementary, rather than independent, diagnostic tests. In the near future, however, they may evolve into primary biomarkers which are used in the early stages of acute coronary syndrome management, when the enzyme levels in the bloodstream are still low and undetectable [116].

The development and execution of well-designed clinical trials to implement the molecular knowledge of multiple validated biomarkers per patient is required, and especially in CVDs, due to the various contributing risk factors and the heterogeneity observed in their pathophysiology. It is noteworthy to mention that only a very limited prospective use of genomic biomarkers in clinical trials related to new drug development as CVD therapeutics has been shown to exist, although there is genomic evidence for the investigated drug targets [179]. It has also been shown that the biomarker application in clinical trials of cardiovascular new drug development is much smaller than in other medical specialties such as oncology. Moreover, the inconsistences identified upon the use of biomarkers in clinical trials of CVDs have recently sparked discussions about the creation of international guidance on quality assurance in the selection and reliability of biomarkers, sample type, collection times, analytical methods, and storage for future research [193]. Complementary to this, lack of supportive cost-effectiveness of pharmacogenomics implementation in the everyday care of CVDs has been identified, thus calling for further improvement of clinical trial environment [194]. This shortcoming clearly highlights the gap between the design and execution of clinical trials, and the exploitation of genetic knowledge for the establishment of pharmacogenomics-guided decisions in the therapy of CVDs and the advancement of precision medicine. Moreover, the combinatorial nature of the factors modulating the genome function in physiology also seemingly contributes to CVDs’ pathophysiology. To this end, we recently published the design and execution of a prospective clinical study lying within the previously discussed concept of pharmacogenomics and precision medicine [52]. In particular, the design of this trial (GESS trial; ClinicalTrials.gov Identifier: NCT03150680), in addition to clinical, epidemiological and laboratory variables, was to include in each enrolled patient a targeted genomic analysis that covers 228 SNP biomarkers in a 117-gene network of pathophysiological and pharmacogenomic relevance. The severity of CAD in a real-world setting of patients undergoing coronary angiography was the primary rationale of the study, confirming the impact of the patients’ genomic profile in the susceptibility of CAD and the response to clopidogrel and statin therapy [52].

Nowadays, the availability of approaches for big health data analysis and the challenge of artificial intelligence methodologies offer hope for the effective treatment of complex classification, clustering, and predictive modeling tasks in cardiovascular research. Consequently, the handling and monitoring of CVD patients has been greatly benefited from such algorithms and practices based on artificial intelligence [195,196,197]. These efforts are parallel to the current healthcare environment, where the power in computerized systems allows the clinical implementation of data-driven machine learning platforms to guide therapeutic decisions in the clinical setting. Recently, by utilizing data from the GESS trial, we have developed a risk-stratification machine learning framework for the prediction of coronary artery disease severity. This data-driven predictive model, through the additional use of pharmacogenomics information, could additionally contribute to a more personalized therapy management and handling of CVD patients [198]. Overall, the use of machine learning platforms and the continuous development of relevant algorithms which are applied to predictive modeling obviously help practitioners to improve their practice and advance the progression of precision cardiovascular medicine.

Nonetheless, novel individualized treatments should be developed based on the existing knowledge of miRNAs. The manipulation of miRNAs and thus gene expression provides a revolutionary chance to block or even reverse the progression of atherosclerosis. However, a continuing restriction of miRNA-based therapies is the difficulty in achieving the steady, targeted delivery of pharmacological compounds to miRNA targets. Although the emergence of miRNA therapeutics has not yet been translated into FDA-approved candidates for medical intervention, candidate drugs are in clinical development, or in phase 1 and phase 2 clinical trials [199]. Hopefully, the merging of miRNA and nanomedicine technology will help in the translation of both of these powerful therapeutic tools, and will facilitate their integration into the daily clinical routine. In any case, however, by building infrastructures in the clinical setting of CVDs which are capable, in real-time, of implementing molecular knowledge, of contributing to interdisciplinary translational research efforts, of providing suitable registries and clinical databases, and of encouraging and facilitating the education of practitioners with synergies, the scientific society is expected to lead to rigorous and significant advancements in cardiovascular precision medicine and pharmacogenomics in the years to come.

## Figures and Tables

**Table 1:** Top 10 miRNAs implicated in CVDs.

	Mature_Mirna	Targeted Genes	SNPs	Drugs	Disease_Drug
1	miR-155-5p	241	5	15	Cardiac hypertrophy; hypertension
2	miR-21-5p	153	4	11	Myocardial infarction; heart failure; vascular disease; cardiac hypertrophy; cardiomyopathy, dilated; stroke
3	miR-145-5p	151	3	9	Vascular disease; supravalvar aortic stenosis
4	miR-34a-5p	148	4	31	Stroke
5	miR-125b-5p	126	5	4	Cardiac hypertrophy; heart failure; vascular disease; cardiomyopathy, dilated; supravalvar aortic stenosis; cardiovascular; cardiomyopathy, idiopathic dilated; stroke
6	miR-29a-3p	123	7	28	Cardiac hypertrophy; cardiomyopathy, dilated; stroke
7	miR-24-3p	110	4	1	Cardiac hypertrophy; heart failure; cardiovascular; cardiomyopathy, dilated; supravalvar aortic stenosis; stroke
8	miR-29b-3p	109	6	15	Cardiac hypertrophy; myocardial infarction; cardiomyopathy, dilated; stroke
9	miR-200c-3p	98	3	5	Cardiomyopathy, dilated
10	miR-17-5p	95	3	8	Cardiomyopathy, dilated; stroke

**Table 2:** Top 10 miRNAs implicated in atherosclerosis, cardiomyopathy and lipid metabolism disorder.

	Atherosclerosis		Cardiomyopathy		Lipid Metabolism Disorder	
	Mature_Mirna	Targeted Genes	Mature_Mirna	Targeted Genes	Mature_Mirna	Targeted Genes
1	miR-146a-5p *	18	miR-21-5p	10	miR-138-5p *	8
2	miR-155-5p	15	miR-24-3p	9	miR-146a-5p *	6
3	miR-21-5p	15	miR-145-5p	7	miR-26a-5p *	5
4	miR-24-3p	15	miR-138-5p *	7	miR-27a-3p *	5
5	miR-29b-3p	13	miR-143-3p *	7	miR-145-5p	5
6	miR-143-3p *	12	miR-17-5p	7	miR-130a-3p *	5
7	miR-145-5p	12	miR-155-5p	6	miR-98-5p *	5
8	miR-221-3p *	12	miR-125b-5p	6	miR-130b-3p *	5
9	miR-126-3p *	11	miR-146a-5p *	6	miR-223-3p *	4
10	miR-138-5p *	11	miR-133b *	5	miR-1-3p *	4

* miRNAs not listed in top 10 miRs implicated in CVDs (Table 1).

## Supplementary Materials

The following supporting information can be downloaded at: https://www.mdpi.com/article/10.3390/cells11040607/s1. Table S1: multiMiR_CAD_miRNAs_results.xlsx; Table S2: DOSE_terms_enrichment_results.xlsx; Table S3: Reactome_pathways_enrichment_results.xlsx; Table S4: List of selected validated target genes (VTGs) with their interacting miRs.
