# Supplementary material for: Dissecting miRNA–Gene Networks to Map Clinical Utility Roads of Pharmacogenomics-Guided Therapeutic Decisions in Cardiovascular Precision Medicine
Source: Cells. 2022 Feb 10;11(4):607. doi: 10.3390/cells11040607 (PMC8870388; doi:10.3390/cells11040607)
Supplement: Supplementary file 1 [file cells-11-00607-s001.zip › Suppl.Table_S4_List of selected VTGs_miRs.pdf]

Table S4. List of selected validated target genes (VTGs) with interacted miRs.

Gene	miR-	Validation method	References
**VEGFA**			
	miR-1-3p	EL, IHC, IFA, luc., MA, qPCR, WB	[1]–[3]
	miR-101-3p	luc., qPCR	[4], [5]
	miR-101-5p	luc., qPCR, WB	[6]
	miR-106a-5p	EL, luc.	[7]
	miR-106b-5p	EL, luc.	[7]
	miR-107	EL, luc.	[7]
	miR-125a-3p	IHC, Luc, qPCR, WB	[8]
	miR-125a-5p	EL, Luc, qPCR, WB	[7], [8]
	miR-126-3p	Luc, WB, qPCR	[9]–[14]
	miR-126-5p	Luc	[15]
	miR-133a-3p	Reporter assay (Non-Functional MTI)	[10]
	miR-140-5p	EL, Luc, qPCR, WB	[7], [16], [17]
	miR-145-5p	IHC, Luc, NB, qPCR, WB	[18]–[20]
	miR-150-5p	EL, Luc	[7]
	miR-15a-5p	ChIP, IHC, Luc, MA, qPCR, WB, EL	[7], [21]–[24]
	miR-15b-5p	EL, Luc	[7]
	miR-16-5p	Luc, qPCR, WB, EL	[7], [21], [22], [25]–[27]
	miR-17-5p	EL, Luc	[7]
	miR-181a-5p	MA, qPCR, EL (Non-Functional MTI)	[28]
	miR-185-5p	Luc, WB	[29]
	miR-186-5p	ChIP, WB, IHC, Luc, MA, qPCR	[23]
	miR-195-5p	EL, Luc	[7], [30], [31]
	miR-199a-3p	EL, Luc, qPCR, WB	[32]
	miR-199a-5p	Luc, MA, qPCR, WB	[33]–[35]
	miR-200c-3p	Luc, WB	[36], [37]
	miR-205-3p	EL, Luc, qPCR, WB	[38]
	miR-205-5p	EL, Flow, Luc, qPCR, WB	[39]–[42]
	miR-20a-5p	EL, Luc	[7], [43]
	miR-20b-5p	EL, Luc, qPCR, WB	[7], [44]
	miR-21-5p	WB, qPCR	[45]
	miR-296-5p	qPCR, WB	[46]
	miR-29a-3p	IHC, Luc, qPCR, WB	[47], [48]
	miR-29b-3p	EL, IHC, Luc, qPCR, WB	[7], [49], [50]
	miR-29c-3p	Flow, IHC, Luc, qPCR, WB	[47]
	miR-34a-5p	EL, Luc (Non-Functional MTI)	[7]
	miR-361-5p	EL, Luc, qPCR, WB	[7], [51], [52]
	miR-372-3p	EL, Luc	[7]
	miR-373-3p	EL, Luc	[7]
	miR-378a-3p	Luc, qPCR, WB, EL	[7], [53]
	miR-429	Luc	[54]
	miR-520h	EL, Luc	[7]
	hsa-miR-93-5p	Luc, qPCR, WB, EL	[7], [55]
**AGTR1**			
	miR-155-5p	Luc, qPCR, NB, WB, QPA	[56]–[59]
	miR-34a-5p	Luc, qPCR, WB	[60]
**HMGCR**			

	miR-27a-3p	Luc, qPCR, NGS	[61]
	miR-29a-3p	Luc, qPCR, WB	[62], [63]
	miR-29b-3p	Luc, qPCR, WB	[63]
	miR-29c-3p	Luc, qPCR, WB	[63]
**ITGB3**			
	let-7a-5p	Luc, qPCR, WB	[64]
	let-7c-5p	Luc, qPCR, WB, ICC	[65]
	miR-17-3p	Luc, qPCR, WB	[66]
	miR-30a-5p	Luc, WB	[67]
	miR-30c-5p	qPCR [Functional MTI (Weak)]	[68]
	miR-98-5p	Luc, qPCR, WB	[69]
**PPARG**			
	miR-130a-3p	EL, IF, Luc, MA, qPCR, WB, ChIP, GFP	[70]–[73]
	miR-130b-3p	Luc, qPCR, WB, GFP	[70], [74], [75]
	miR-138-5p	qPCR [Functional MTI (Weak)]	[76]
	miR-20a-5p	Luc	[77]
	miR-20b-5p	Luc, qPCR	[78]
	miR-27a-3p	Luc, qPCR, WB	[79], [80]
	miR-27b-3p	Luc, qPCR, WB	[81]–[83]
**Tp53**			
	hsa-miR-34a-5p	Luc, MA, NB, qPCR, NGS, IHC, ICC, Fl, WB	[84]–[87]
	hsa-miR-106b-5p	qPCR [Functional MTI (Weak)]	[88]
	hsa-miR-300	Luc, qPCR, WB, IP	[89]–[91]
	hsa-miR-638	Luc, qPCR, WB	[92]
	hsa-miR-377-3p	Luc, qPCR, WB	[93]
	hsa-miR-28-5p	Luc	[94]
	hsa-miR-125b-5p	Luc, qPCR, WB, NGS	[95]–[100]
	hsa-miR-10b-5p	Luc	[101]
	hsa-miR-30e-5p	Luc, qPCR, WB	[102]
	hsa-miR-30b-5p	Luc, qPCR, WB	[102]
	hsa-miR-30d-5p	Luc, qPCR, WB	[102], [103]
	hsa-miR-221-3p	WB (Non-Functional MTI)	[104]
	hsa-miR-222-3p	WB (Non-Functional MTI)	[104]
	hsa-miR-214-3p	Luc	[105], [106]
	hsa-miR-150-5p	Luc, WB, qPCR	[107], [108]
	hsa-miR-125a-5p	Luc, NB, qPCR, WB	[109], [110]
	hsa-miR-16-5p	Luc, WB	[111]
	hsa-miR-19b-3p	IHC, Luc, qPCR, WB	[112]
	hsa-miR-20a-5p	qPCR [Functional MTI (Weak)]	[88]
	hsa-miR-18a-5p	qPCR [Functional MTI (Weak)]	[88]
	hsa-miR-485-5p	qPCR [Non-Functional MTI (Weak)]	[113]
	hsa-miR-491-5p	Fl, GFP, qPCR, WB	[114]
	hsa-miR-151a-5p	ChIP, Luc, qPCR, WB	[115]
	hsa-miR-27a-3p	Luc	[116]
	hsa-miR-30a-5p	Luc, qPCR, WB	[102]
	hsa-miR-106a-5p	qPCR [Functional MTI (Weak)]	[88]
	hsa-miR-150-3p	qPCR, WB	[117]
	hsa-miR-30c-5p	Luc, GFP, NGS, Fl, NB, qPCR, WB	[102], [118]

hsa-miR-28-3p	Luc	[94]
hsa-miR-24-3p	IHC, Luc, qPCR, WB	[119]
hsa-miR-214-5p	qPCR, WB	[120]
hsa-miR-15a-5p	Luc, WB	[111]
hsa-miR-17-5p	qPCR [Functional MTI (Weak)]	[88]
hsa-miR-25-3p	Luc, qPCR, WB	[103]
**EGFR**		
hsa-miR-133a-3p	Fl, Luc, qPCR, WB	[121]–[125]
hsa-miR-133b	Fl, ISH, Luc, qPCR, WB	[123], [126], [127]
hsa-miR-491-5p	Luc, qPCR, WB	[128], [129]
hsa-miR-7-5p	IHC, Luc, MA, qPCR, WB, Fl, ISH	[130]–[139]
hsa-miR-146a-5p	IHC, Luc, qPCR, WB	[140]–[147]
hsa-miR-7-1-3p	MA, qPCR, WB	[148]
hsa-miR-125b-5p	IHC, WB	[149]
hsa-miR-21-5p	Luc, MA, WB	[137], [150]
hsa-miR-574-3p	Luc, MA, qPCR, WB	[151]
hsa-let-7a-5p	Luc	[152]
hsa-miR-27a-5p	WB	[153]
hsa-miR-218-5p	Luc, qPCR, WB	[154], [155]
hsa-miR-146b-5p	Luc, qPCR, WB	[146]
hsa-miR-135a-5p	Luc, WB	[156]
hsa-miR-125a-5p	qPCR, WB	[157]
hsa-miR-27a-3p	Luc, qPCR, WB	[158], [159]
hsa-miR-145-5p	MA, qPCR, WB	[160]
hsa-miR-132-3p	IHC, Luc, qPCR, WB	[161]
hsa-miR-27b-3p	qPCR, Luc, WB	[162]
hsa-miR-302b-3p	Luc, qPCR, WB	[163]

IHC: immunohistochemistry; IF: immunofluorescence; luc: luciferase reporter assay; qPCR: quantitative real-time PCR; WB: western blotting analysis; EL: ELISA; MA: Microarray; NB: Northern blot; ChIP: Chromatin immunoprecipitation; MTI: miRNA - target interaction; QPA: Quantitative proteomic approach; NGS: Next Generation Sequencing; ICC: Immunocytochemistry; GFP: GFP reporter assay reporter assay; IP: Immunoprecipitation; Fl: Flow cytometry; ISH: In situ hybridization

References

[1] M. Xie *et al.*, “MicroRNA-1 acts as a tumor suppressor microRNA by inhibiting angiogenesis-related growth factors in human gastric cancer,” *Gastric Cancer*, vol. 21, no. 1, pp. 41–54, Jan. 2018, doi: 10.1007/s10120-017-0721-x.

[2] J. Niu, Y. Sun, Q. Guo, D. Niu, and B. Liu, “miR-1 Inhibits Cell Growth, Migration, and Invasion by Targeting VEGFA in Osteosarcoma Cells,” *Disease Markers*, vol. 2016, pp. 1–8, 2016, doi: 10.1155/2016/7068986.

[3] H. Shen *et al.*, “Reprogramming of Normal Fibroblasts into Cancer-Associated Fibroblasts by miRNAs-Mediated CCL2/VEGFA Signaling,” *PLoS Genetics*, vol. 12, no. 8, Aug. 2016, doi: 10.1371/journal.pgen.1006244.

[4] Y. Lei *et al.*, “miR-101 Suppresses Vascular Endothelial Growth Factor C That Inhibits Migration and Invasion and Enhances Cisplatin Chemosensitivity of Bladder Cancer Cells,” *PLOS ONE*, vol. 10, no. 2, p. e0117809, Feb. 2015, doi: 10.1371/journal.pone.0117809.

[5] J. Zhang, C. Han, H. Zhu, K. Song, and T. Wu, “miR-101 Inhibits Cholangiocarcinoma Angiogenesis through Targeting Vascular Endothelial Growth Factor (VEGF),” *The American Journal of Pathology*, vol. 182, no. 5, pp. 1629–1639, May 2013, doi: 10.1016/j.ajpath.2013.01.045.

[6] Z. LIU, J. WANG, Y. MAO, B. ZOU, and X. FAN, “MicroRNA-101 suppresses migration and invasion via targeting vascular endothelial growth factor-C in hepatocellular carcinoma cells,” *Oncology Letters*, vol. 11, no. 1, pp. 433–438, Jan. 2016, doi: 10.3892/ol.2015.3832.

[7] W. Ye *et al.*, “The effect of central loops in miRNA: MRE duplexes on the efficiency of miRNA-mediated gene regulation,” *PLoS ONE*, vol. 3, no. 3, Mar. 2008, doi: 10.1371/journal.pone.0001719.

[8] J. DAI, J. WANG, L. YANG, Y. XIAO, and Q. RUAN, “miR-125a regulates angiogenesis of gastric cancer by targeting vascular endothelial growth factor A,” *International Journal of Oncology*, vol. 47, no. 5, pp. 1801–1810, Nov. 2015, doi: 10.3892/ijo.2015.3171.

[9] B. Liu, X.-C. Peng, X.-L. Zheng, J. Wang, and Y.-W. Qin, “MiR-126 restoration down-regulate VEGF and inhibit the growth of lung cancer cell lines in vitro and in vivo,” *Lung Cancer*, vol. 66, no. 2, pp. 169–175, Nov. 2009, doi: 10.1016/j.lungcan.2009.01.010.

[10] N. Zhu *et al.*, “Endothelial-specific intron-derived miR-126 is down-regulated in human breast cancer and targets both VEGFA and PIK3R2,” *Molecular and Cellular Biochemistry*, vol. 351, no. 1–2, pp. 157–164, May 2011, doi: 10.1007/s11010-011-0723-7.

[11] X. Zhu *et al.*, “miR-126 enhances the sensitivity of non-small cell lung cancer cells to anticancer agents by targeting vascular endothelial growth factor A,” *Acta Biochimica et Biophysica Sinica*, vol. 44, no. 6, pp. 519–526, Jun. 2012, doi: 10.1093/abbs/gms026.

[12] Y. ZHANG *et al.*, “Epigenetic silencing of miR-126 contributes to tumor invasion and angiogenesis in colorectal cancer,” *Oncology Reports*, vol. 30, no. 4, pp. 1976–1984, Oct. 2013, doi: 10.3892/or.2013.2633.

[13] H. Chen *et al.*, “Reduced miR-126 expression facilitates angiogenesis of gastric cancer through its regulation on VEGF-A,” *Oncotarget*, vol. 5, no. 23, pp. 11873–11885, Dec. 2014, doi: 10.18632/oncotarget.2662.

[14] C.-J. Guo *et al.*, “Dynamic expression of miR-126 ∗ and its effects on proliferation and contraction of hepatic stellate cells,” *FEBS Letters*, vol. 587, no. 23, pp. 3792–3801, Nov. 2013, doi: 10.1016/j.febslet.2013.09.047.

[15] R. Tang, L. Pei, T. Bai, and J. Wang, “Down-regulation of microRNA-126-5p contributes to overexpression of VEGFA in lipopolysaccharide-induced acute lung injury,” *Biotechnology Letters*, vol. 38, no. 8, pp. 1277–1284, Aug. 2016, doi: 10.1007/s10529-016-2107-2.

[16] W. Zhang *et al.*, “MicroRNA-140-5p Inhibits the Progression of Colorectal Cancer by Targeting VEGFA,” *Cellular Physiology and Biochemistry*, vol. 37, no. 3, pp. 1123–1133, 2015, doi: 10.1159/000430237.

[17] J. SUN, S. TAO, L. LIU, D. GUO, Z. XIA, and M. HUANG, “miR-140-5p regulates angiogenesis following ischemic stroke by targeting VEGFA,” *Molecular Medicine Reports*, vol. 13, no. 5, pp. 4499–4505, May 2016, doi: 10.3892/mmr.2016.5066.

[18] C. Zou *et al.*, “MiR-145 inhibits tumor angiogenesis and growth by N-RAS and VEGF,” *Cell Cycle*, vol. 11, no. 11, pp. 2137–2145, Jun. 2012, doi: 10.4161/cc.20598.

[19] L. Fan, Q. Wu, X. Xing, Y. Wei, and Z. Shao, “MicroRNA-145 targets vascular endothelial growth factor and inhibits invasion and metastasis of osteosarcoma cells,” *Acta Biochimica et Biophysica Sinica*, vol. 44, no. 5, pp. 407–414, May 2012, doi: 10.1093/abbs/gms019.

[20] S. Wu *et al.*, “Argonaute 2 promotes myeloma angiogenesis via microRNA dysregulation,” *Journal of Hematology & Oncology*, vol. 7, no. 1, p. 40, Dec. 2014, doi: 10.1186/1756-8722-7-40.

[21] G. Spinetti *et al.*, “MicroRNA-15a and MicroRNA-16 Impair Human Circulating Proangiogenic Cell Functions and Are Increased in the Proangiogenic Cells and Serum of Patients With Critical Limb Ischemia,” *Circulation Research*, vol. 112, no. 2, pp. 335–346, Jan. 2013, doi: 10.1161/CIRCRESAHA.111.300418.

[22] C.-Y. Sun *et al.*, “miR-15a and miR-16 affect the angiogenesis of multiple myeloma by targeting VEGF,” *Carcinogenesis*, vol. 34, no. 2, pp. 426–435, Feb. 2013, doi: 10.1093/carcin/bgs333.

[23] E. Terzuoli *et al.*, “Linking microsomal prostaglandin E Synthase-1/PGE-2 pathway with miR-15a and −186 expression: Novel mechanism of VEGF modulation in prostate cancer,” *Oncotarget*, vol. 7, no. 28, pp. 44350–44364, Jul. 2016, doi: 10.18632/oncotarget.10051.

[24] H. Chen and Y. Tian, “MiR-15a-5p regulates viability and matrix degradation of human osteoarthritis chondrocytes via targeting VEGFA,” *BioScience Trends*, vol. 10, no. 6, pp. 482–488, 2016, doi: 10.5582/bst.2016.01187.

[25] Z. S. Karaa, J. S. Iacovoni, A. Bastide, E. Lacazette, C. Touriol, and H. Prats, “The VEGF IRESes are differentially susceptible to translation inhibition by miR-16,” *RNA*, vol. 15, no. 2, pp. 249–254, Feb. 2009, doi: 10.1261/rna.1301109.

[26] A. Chamorro-Jorganes, E. Araldi, L. O. F. Penalva, D. Sandhu, C. Fernández-Hernando, and Y. Suárez, “MicroRNA-16 and MicroRNA-424 Regulate Cell-Autonomous Angiogenic Functions in Endothelial Cells via Targeting Vascular Endothelial Growth Factor Receptor-2 and Fibroblast Growth Factor Receptor-1,” *Arteriosclerosis, Thrombosis, and Vascular Biology*, vol. 31, no. 11, pp. 2595–2606, Nov. 2011, doi: 10.1161/ATVBAHA.111.236521.

[27] Y. Wang *et al.*, “miR-16 inhibits the proliferation and angiogenesis-regulating potential of mesenchymal stem cells in severe pre-eclampsia,” *FEBS Journal*, vol. 279, no. 24, pp. 4510–4524, Dec. 2012, doi: 10.1111/febs.12037.

[28] X. Sun, L. Wei, Q. Chen, and R. M. Terek, “MicroRNA Regulates Vascular Endothelial Growth Factor Expression in Chondrosarcoma Cells,” *Clinical Orthopaedics & Related Research*, vol. 473, no. 3, pp. 907–913, Mar. 2015, doi: 10.1007/s11999-014-3842-0.

[29] R. Wang *et al.*, “MiR-185 is involved in human breast carcinogenesis by targeting Vegfa,” *FEBS Letters*, vol. 588, no. 23, pp. 4438–4447, Nov. 2014, doi: 10.1016/j.febslet.2014.09.045.

[30] M. Wang, J. Zhang, L. Tong, X. Ma, and X. Qiu, “MiR-195 is a key negative regulator of hepatocellular carcinoma metastasis by targeting FGF2 and VEGFA.,” *International journal of clinical and experimental pathology*, vol. 8, no. 11, pp. 14110–20, 2015, [Online]. Available: http://www.ncbi.nlm.nih.gov/pubmed/26823724

[31] R. Wang *et al.*, “MicroRNA-195 Suppresses Angiogenesis and Metastasis of Hepatocellular Carcinoma by Inhibiting the Expression of VEGF, VAV2, and CDC42,” *Hepatology*, vol. 58, no. 2, pp. 642–653, Aug. 2013, doi: 10.1002/hep.26373.

[32] A. Ghosh *et al.*, “MiRNA199a-3p suppresses tumor growth, migration, invasion and angiogenesis in hepatocellular carcinoma by targeting VEGFA, VEGFR1, VEGFR2, HGF and MMP2,” *Cell Death & Disease*, vol. 8, no. 3, pp. e2706–e2706, Mar. 2017, doi: 10.1038/cddis.2017.123.

[33] K. Morita *et al.*, “Relevance of microRNA-18a and microRNA-199a-5p to hepatocellular carcinoma recurrence after living donor liver transplantation,” *Liver Transplantation*, vol. 22, no. 5, pp. 665–676, May 2016, doi: 10.1002/lt.24400.

[34] C.-Y. Hsu *et al.*, “miRNA-199a-5p regulates VEGFA in endometrial mesenchymal stem cells and contributes to the pathogenesis of endometriosis,” *The Journal of Pathology*, vol. 232, no. 3, pp. 330–343, Feb. 2014, doi: 10.1002/path.4295.

[35] L. Dai, W. Lou, J. Zhu, X. Zhou, and W. Di, “MiR-199a inhibits the angiogenic potential of endometrial stromal cells under hypoxia by targeting HIF-1α/VEGF pathway.,” *International journal of clinical and experimental pathology*, vol. 8, no. 5, pp. 4735–44, 2015, [Online]. Available: http://www.ncbi.nlm.nih.gov/pubmed/26191163

[36] H. Panda, L. Pelakh, T.-D. Chuang, X. Luo, O. Bukulmez, and N. Chegini, “Endometrial miR-200c is Altered During Transformation into Cancerous States and Targets the Expression of ZEBs , VEGFA , FLT1 , IKKβ , KLF9 , and FBLN5,” *Reproductive Sciences*, vol. 19, no. 8, pp. 786–796, Aug. 2012, doi: 10.1177/1933719112438448.

[37] T.-D. Chuang, H. Panda, X. Luo, and N. Chegini, “miR-200c is aberrantly expressed in leiomyomas in an ethnic-dependent manner and targets ZEBs, VEGFA, TIMP2, and FBLN5,” *Endocrine-Related Cancer*, vol. 19, no. 4, pp. 541–556, Aug. 2012, doi: 10.1530/ERC-12-0007.

[38] Y. Hu, Y. Qiu, E. Yagüe, W. Ji, J. Liu, and J. Zhang, "miRNA-205 targets VEGFA and FGF2 and regulates resistance to chemotherapeutics in breast cancer," *Cell Death & Disease*, vol. 7, no. 6, pp. e2291–e2291, Jun. 2016, doi: 10.1038/cddis.2016.194.
[39] X. YUE *et al.*, "MicroRNA-205 functions as a tumor suppressor in human glioblastoma cells by targeting VEGF-A," *Oncology Reports*, vol. 27, no. 4, pp. 1200–1206, Apr. 2012, doi: 10.3892/or.2011.1588.
[40] R. Zhao, L. Qian, and L. Jiang, "miRNA-dependent cross-talk between VEGF and Ang-2 in hypoxia-induced microvascular dysfunction," *Biochemical and Biophysical Research Communications*, vol. 452, no. 3, pp. 428–435, Sep. 2014, doi: 10.1016/j.bbrc.2014.08.096.
[41] H. Wu, S. Zhu, and Y.-Y. Mo, "Suppression of cell growth and invasion by miR-205 in breast cancer," *Cell Research*, vol. 19, no. 4, pp. 439–448, Apr. 2009, doi: 10.1038/cr.2009.18.
[42] Y. Li *et al.*, "miR-205 suppresses the proliferative and migratory capacity of human osteosarcoma MG-63 cells by targeting VEGFA," *OncoTargets and Therapy*, p. 2635, Sep. 2015, doi: 10.2147/OTT.S80088.
[43] H. Ma *et al.*, "MicroRNA-17~92 inhibits colorectal cancer progression by targeting angiogenesis," *Cancer Letters*, vol. 376, no. 2, pp. 293–302, Jul. 2016, doi: 10.1016/j.canlet.2016.04.011.
[44] T. Xue *et al.*, "Clinicopathological Significance of MicroRNA-20b Expression in Hepatocellular Carcinoma and Regulation of HIF-1 α and VEGF Effect on Cell Biological Behaviour," *Disease Markers*, vol. 2015, pp. 1–10, 2015, doi: 10.1155/2015/325176.
[45] L.-Z. Liu *et al.*, "MiR-21 Induced Angiogenesis through AKT and ERK Activation and HIF-1α Expression," *PLoS ONE*, vol. 6, no. 4, p. e19139, Apr. 2011, doi: 10.1371/journal.pone.0019139.
[46] J. FENG *et al.*, "Pro-angiogenic microRNA-296 upregulates vascular endothelial growth factor and downregulates Notch1 following cerebral ischemic injury," *Molecular Medicine Reports*, vol. 12, no. 6, pp. 8141–8147, Dec. 2015, doi: 10.3892/mmr.2015.4436.
[47] H. Zhang *et al.*, "Cell-derived microvesicles mediate the delivery of miR-29a/c to suppress angiogenesis in gastric carcinoma," *Cancer Letters*, vol. 375, no. 2, pp. 331–339, Jun. 2016, doi: 10.1016/j.canlet.2016.03.026.
[48] L. Chen *et al.*, "miR-29a suppresses growth and invasion of gastric cancer cells in vitro by targeting VEGF-A," *BMB Reports*, vol. 47, no. 1, pp. 39–44, Jan. 2014, doi: 10.5483/BMBRep.2014.47.1.079.
[49] H.-X. Chen, X.-X. Xu, B.-Z. Tan, Z. Zhang, and X.-D. Zhou, "MicroRNA-29b Inhibits Angiogenesis by Targeting VEGFA through the MAPK/ERK and PI3K/Akt Signaling Pathways in Endometrial Carcinoma," *Cellular Physiology and Biochemistry*, vol. 41, no. 3, pp. 933–946, 2017, doi: 10.1159/000460510.
[50] J. Chou, J. H. Lin, A. Brenot, J. Kim, S. Provot, and Z. Werb, "GATA3 suppresses metastasis and modulates the tumour microenvironment by regulating microRNA-29b expression," *Nature Cell Biology*, vol. 15, no. 2, pp. 201–213, Feb. 2013, doi: 10.1038/ncb2672.
[51] A. Kanitz *et al.*, "The Expression Levels of MicroRNA-361-5p and Its Target VEGFA Are Inversely Correlated in Human Cutaneous Squamous Cell Carcinoma," *PLoS ONE*, vol. 7, no. 11, p. e49568, Nov. 2012, doi: 10.1371/journal.pone.0049568.
[52] M. Dal Monte, D. Landi, D. Martini, and P. Bagnoli, "Antiangiogenic role of miR-361 in human umbilical vein endothelial cells: functional interaction with the peptide somatostatin," *Naunyn-Schmiedeberg's Archives of Pharmacology*, vol. 386, no. 1, pp. 15–27, Jan. 2013, doi: 10.1007/s00210-012-0808-1.
[53] H. Deng *et al.*, "MicroRNA-195 and microRNA-378 mediate tumor growth suppression by epigenetical regulation in gastric cancer," *Gene*, vol. 518, no. 2, pp. 351–359, Apr. 2013, doi: 10.1016/j.gene.2012.12.103.
[54] D. CHEN *et al.*, "Tumor suppressive microRNA-429 regulates cellular function by targeting VEGF in clear cell renal cell carcinoma," *Molecular Medicine Reports*, vol. 13, no. 2, pp. 1361–1366, Feb. 2016, doi: 10.3892/mmr.2015.4653.
[55] X. Lv, P. Chen, and W. Liu, "Down regulation of MiR-93 contributes to endometriosis through targeting MMP3 and VEGFA.," *American journal of cancer research*, vol. 5, no. 5, pp. 1706–17, 2015, [Online]. Available: http://www.ncbi.nlm.nih.gov/pubmed/26175939
[56] P. Sethupathy *et al.*, "Human microRNA-155 on chromosome 21 differentially interacts with its polymorphic target in the AGTR1 3′ untranslated region: a mechanism for functional single-nucleotide polymorphisms related to phenotypes.," *American journal of human genetics*, vol. 81, no. 2, pp. 405–13, Aug. 2007, doi: 10.1086/519979.
[57] J. H. Gibcus *et al.*, "Hodgkin lymphoma cell lines are characterized by a specific miRNA expression profile.," *Neoplasia (New York, N.Y.)*, vol. 11, no. 2, pp. 167–76, Feb. 2009, doi: 10.1593/neo.08980.

[58] N. Zhu *et al.*, “Endothelial enriched microRNAs regulate angiotensin II-induced endothelial inflammation and migration,” *Atherosclerosis*, vol. 215, no. 2, pp. 286–293, Apr. 2011, doi: 10.1016/j.atherosclerosis.2010.12.024.
[59] U. Haas, G. Sczakiel, and S. D. Laufer, “MicroRNA-mediated regulation of gene expression is affected by disease-associated SNPs within the 3′-UTR via altered RNA structure,” *RNA Biology*, vol. 9, no. 6, pp. 924–937, Jun. 2012, doi: 10.4161/rna.20497.
[60] Y. Pu *et al.*, “The miR-34a-5p promotes the multi-chemoresistance of osteosarcoma via repression of the AGTR1 gene.,” *BMC cancer*, vol. 17, no. 1, p. 45, 2017, doi: 10.1186/s12885-016-3002-x.
[61] S. R. Selitsky *et al.*, “Transcriptomic analysis of chronic hepatitis b and c and liver cancer reveals microrna-mediated control of cholesterol synthesis programs,” *mBio*, vol. 6, no. 6, Dec. 2015, doi: 10.1128/mBio.01500-15.
[62] C. L. Kurtz, E. E. Fannin, C. L. Toth, D. S. Pearson, K. C. Vickers, and P. Sethupathy, “Inhibition of miR-29 has a significant lipid-lowering benefit through suppression of lipogenic programs in liver,” *Scientific Reports*, vol. 5, no. 1, pp. 1–13, Aug. 2015, doi: 10.1038/srep12911.
[63] M. X. Liu *et al.*, “Dicer1/miR-29/HMGCR axis contributes to hepatic free cholesterol accumulation in mouse non-alcoholic steatohepatitis,” *Acta Pharmacologica Sinica*, vol. 38, no. 5, pp. 660–671, Jan. 2017, doi: 10.1038/aps.2016.158.
[64] D. W. Müller and A.-K. Bosserhoff, “Integrin β3 expression is regulated by let-7a miRNA in malignant melanoma,” *Oncogene*, vol. 27, no. 52, pp. 6698–6706, Nov. 2008, doi: 10.1038/onc.2008.282.
[65] B. Zhao *et al.*, “MicroRNA let-7c inhibits migration and invasion of human non-small cell lung cancer by targeting ITGB3 and MAP4K3,” *Cancer Letters*, vol. 342, no. 1, pp. 43–51, Jan. 2014, doi: 10.1016/j.canlet.2013.08.030.
[66] C. Gong, Z. Yang, F. Wu, L. Han, Y. Liu, and W. Gong, “miR-17 inhibits ovarian cancer cell peritoneal metastasis by targeting ITGA5 and ITGB1,” *Oncology Reports*, vol. 36, no. 4, pp. 2177–2183, Oct. 2016, doi: 10.3892/or.2016.4985.
[67] W. Wei *et al.*, “MiR-30a-5p Suppresses Tumor Metastasis of Human Colorectal Cancer by Targeting ITGB3,” *Cellular Physiology and Biochemistry*, vol. 39, no. 3, pp. 1165–1176, 2016, doi: 10.1159/000447823.
[68] X. Zhang, M. Daucher, D. Armistead, R. Russell, and S. Kottilil, “MicroRNA Expression Profiling in HCV-Infected Human Hepatoma Cells Identifies Potential Anti-Viral Targets Induced by Interferon-α,” *PLoS ONE*, vol. 8, no. 2, p. e55733, Feb. 2013, doi: 10.1371/journal.pone.0055733.
[69] J. Wang, R. Ni, and Y. Huang, “miR-98 targets ITGB3 to inhibit proliferation, migration, and invasion of non-small-cell lung cancer,” *OncoTargets and Therapy*, p. 2689, Sep. 2015, doi: 10.2147/OTT.S90998.
[70] E. K. Lee *et al.*, “miR-130 Suppresses Adipogenesis by Inhibiting Peroxisome Proliferator-Activated Receptor Expression,” *Molecular and Cellular Biology*, vol. 31, no. 4, pp. 626–638, Feb. 2011, doi: 10.1128/mcb.00894-10.
[71] L. LIN, H. LIN, L. WANG, B. WANG, X. HAO, and Y. SHI, “miR-130a regulates macrophage polarization and is associated with non-small cell lung cancer,” *Oncology Reports*, vol. 34, no. 6, pp. 3088–3096, Dec. 2015, doi: 10.3892/or.2015.4301.
[72] S. Su *et al.*, “miR-142-5p and miR-130a-3p are regulated by IL-4 and IL-13 and control profibrogenic macrophage program,” *Nature Communications*, vol. 6, no. 1, p. 8523, Dec. 2015, doi: 10.1038/ncomms9523.
[73] Y. Jiao *et al.*, “MicroRNA-130a expression is decreased in Xinjiang Uygur patients with type 2 diabetes mellitus.,” *American journal of translational research*, vol. 7, no. 10, pp. 1984–91, 2015, [Online]. Available: http://www.ncbi.nlm.nih.gov/pubmed/26692941
[74] X. Sheng *et al.*, “MicroRNA-130b promotes cell migration and invasion by targeting peroxisome proliferator-activated receptor gamma in human glioma,” *Biomedicine & Pharmacotherapy*, vol. 76, pp. 121–126, Dec. 2015, doi: 10.1016/j.biopha.2015.10.003.
[75] M. Zhang, Z. Zhou, J. Wang, and S. Li, “MiR-130b promotes obesity associated adipose tissue inflammation and insulin resistance in diabetes mice through alleviating M2 macrophage polarization via repression of PPAR-γ,” *Immunology Letters*, vol. 180, pp. 1–8, Dec. 2016, doi: 10.1016/j.imlet.2016.10.004.
[76] Z. Yang *et al.*, “MicroRNA hsa-miR-138 Inhibits Adipogenic Differentiation of Human Adipose Tissue-Derived Mesenchymal Stem Cells Through Adenovirus EID-1,” *Stem Cells and Development*, vol. 20, no. 2, pp. 259–267, Feb. 2011, doi: 10.1089/scd.2010.0072.
[77] J. F. Zhang *et al.*, “MiRNA-20a promotes osteogenic differentiation of human mesenchymal stem cells by co-regulating BMP signaling,” *RNA Biology*, vol. 8, no. 5, Sep. 2011, doi: 10.4161/rna.8.5.16043.

[78] J. He *et al.*, "miRNA-Mediated Functional Changes through Co-Regulating Function Related Genes," *PLoS ONE*, vol. 5, no. 10, p. e13558, Oct. 2010, doi: 10.1371/journal.pone.0013558.
[79] B.-Y. Kang *et al.*, "Hypoxia Mediates Mutual Repression between microRNA-27a and PPARγ in the Pulmonary Vasculature," *PLoS ONE*, vol. 8, no. 11, p. e79503, Nov. 2013, doi: 10.1371/journal.pone.0079503.
[80] B. Zou, Z. Ge, W. Zhu, Z. Xu, and C. Li, "Persimmon tannin represses 3T3-L1 preadipocyte differentiation via up-regulating expression of miR-27 and down-regulating expression of peroxisome proliferator-activated receptor-γ in the early phase of adipogenesis," *European Journal of Nutrition*, vol. 54, no. 8, pp. 1333–1343, Dec. 2015, doi: 10.1007/s00394-014-0814-9.
[81] M. Karbiener *et al.*, "microRNA miR-27b impairs human adipocyte differentiation and targets PPARγ," *Biochemical and Biophysical Research Communications*, vol. 390, no. 2, pp. 247–251, Dec. 2009, doi: 10.1016/j.bbrc.2009.09.098.
[82] J.-J. Lee, A. Drakaki, D. Iliopoulos, and K. Struhl, "MiR-27b targets PPARγ to inhibit growth, tumor progression and the inflammatory response in neuroblastoma cells," *Oncogene*, vol. 31, no. 33, pp. 3818–3825, Aug. 2012, doi: 10.1038/onc.2011.543.
[83] S. ZHANG *et al.*, "Elevation of miR-27b by HPV16 E7 inhibits PPARγ expression and promotes proliferation and invasion in cervical carcinoma cells," *International Journal of Oncology*, vol. 47, no. 5, pp. 1759–1766, Nov. 2015, doi: 10.3892/ijo.2015.3162.
[84] H. Wang *et al.*, "Aptamer-Dendrimer Bioconjugates for Targeted Delivery of miR-34a Expressing Plasmid and Antitumor Effects in Non-Small Cell Lung Cancer Cells.," *PloS one*, vol. 10, no. 9, p. e0139136, 2015, doi: 10.1371/journal.pone.0139136.
[85] A. Javeri, M. Ghaffarpour, M. F. Taha, and M. Houshmand, "Downregulation of miR-34a in breast tumors is not associated with either p53 mutations or promoter hypermethylation while it correlates with metastasis.," *Medical oncology (Northwood, London, England)*, vol. 30, no. 1, p. 413, Mar. 2013, doi: 10.1007/s12032-012-0413-7.
[86] H. Liu, B. A. French, J. Li, B. Tillman, and S. W. French, "Altered regulation of miR-34a and miR-483-3p in alcoholic hepatitis and DDC fed mice.," *Experimental and molecular pathology*, vol. 99, no. 3, pp. 552–7, Dec. 2015, doi: 10.1016/j.yexmp.2015.09.005.
[87] F. Navarro and J. Lieberman, "miR-34 and p53: New Insights into a Complex Functional Relationship.," *PloS one*, vol. 10, no. 7, p. e0132767, 2015, doi: 10.1371/journal.pone.0132767.
[88] L. Arabi *et al.*, "Upregulation of the miR-17-92 cluster and its two paraloga in osteosarcoma - reasons and consequences.," *Genes & cancer*, vol. 5, no. 1–2, pp. 56–63, Apr. 2014, doi: 10.18632/genesandcancer.6.
[89] L. Wang and P. Yu, "miR-300 promotes proliferation and EMT-mediated colorectal cancer migration and invasion by targeting p53.," *Oncology reports*, vol. 36, no. 6, pp. 3225–3232, Dec. 2016, doi: 10.3892/or.2016.5193.
[90] Z. Shen *et al.*, "The up-regulation of miR-300 in gastric cancer and its effects on cells malignancy.," *International journal of clinical and experimental medicine*, vol. 8, no. 5, pp. 6773–83, 2015, [Online]. Available: http://www.ncbi.nlm.nih.gov/pubmed/26221215
[91] X.-H. Xu, D.-W. Li, H. Feng, H.-M. Chen, and Y.-Q. Song, "MiR-300 regulate the malignancy of breast cancer by targeting p53.," *International journal of clinical and experimental medicine*, vol. 8, no. 5, pp. 6957–66, 2015, [Online]. Available: http://www.ncbi.nlm.nih.gov/pubmed/26221232
[92] Y. Tay, S. M. Tan, F. A. Karreth, J. Lieberman, and P. P. Pandolfi, "Characterization of dual PTEN and p53-targeting microRNAs identifies microRNA-638/Dnm2 as a two-hit oncogenic locus.," *Cell reports*, vol. 8, no. 3, pp. 714–22, Aug. 2014, doi: 10.1016/j.celrep.2014.06.064.
[93] X. Wen, J.-Q. Wu, W. Peng, J.-F. Feng, and J.-H. Tang, "MicroRNA-377 predicts poor clinical outcome of gastric cancer and induces tumorigenesis by targeting multiple tumor-suppressor genes.," *Oncology reports*, vol. 34, no. 1, pp. 203–10, Jul. 2015, doi: 10.3892/or.2015.3981.
[94] M. Girardot *et al.*, "Persistent STAT5 activation in myeloid neoplasms recruits p53 into gene regulation.," *Oncogene*, vol. 34, no. 10, pp. 1323–32, Mar. 2015, doi: 10.1038/onc.2014.60.
[95] X. Yu, D. M. Cohen, and C. S. Chen, "miR-125b Is an adhesion-regulated microRNA that protects mesenchymal stem cells from anoikis.," *Stem cells (Dayton, Ohio)*, vol. 30, no. 5, pp. 956–64, May 2012, doi: 10.1002/stem.1064.
[96] M. T. N. Le *et al.*, "MicroRNA-125b is a novel negative regulator of p53.," *Genes & development*, vol. 23, no. 7, pp. 862–76, Apr. 2009, doi: 10.1101/gad.1767609.

[97] M. T. N. Le *et al.*, "Conserved regulation of p53 network dosage by microRNA-125b occurs through evolving miRNA-target gene pairs.," *PLoS genetics*, vol. 7, no. 9, p. e1002242, Sep. 2011, doi: 10.1371/journal.pgen.1002242.

[98] K. Iida *et al.*, "miR-125b develops chemoresistance in Ewing sarcoma/primitive neuroectodermal tumor.," *Cancer cell international*, vol. 13, no. 1, p. 21, Mar. 2013, doi: 10.1186/1475-2867-13-21.

[99] V. Nagpal *et al.*, "MiR-125b Is Critical for Fibroblast-to-Myofibroblast Transition and Cardiac Fibrosis.," *Circulation*, vol. 133, no. 3, pp. 291–301, Jan. 2016, doi: 10.1161/CIRCULATIONAHA.115.018174.

[100] M. Inui, G. Martello, and S. Piccolo, "MicroRNA control of signal transduction.," *Nature reviews. Molecular cell biology*, vol. 11, no. 4, pp. 252–63, Apr. 2010, doi: 10.1038/nrm2868.

[101] J. Lin, S. Teo, D. H. Lam, K. Jeyaseelan, and S. Wang, "MicroRNA-10b pleiotropically regulates invasion, angiogenicity and apoptosis of tumor cells resembling mesenchymal subtype of glioblastoma multiforme.," *Cell death & disease*, vol. 3, p. e398, Oct. 2012, doi: 10.1038/cddis.2012.134.

[102] J. Wu *et al.*, "Downregulation of microRNA-30 facilitates podocyte injury and is prevented by glucocorticoids.," *Journal of the American Society of Nephrology : JASN*, vol. 25, no. 1, pp. 92–104, Jan. 2014, doi: 10.1681/ASN.2012111101.

[103] M. Kumar *et al.*, "Negative regulation of the tumor suppressor p53 gene by microRNAs.," *Oncogene*, vol. 30, no. 7, pp. 843–53, Feb. 2011, doi: 10.1038/onc.2010.457.

[104] C.-J. Yang *et al.*, "miR-221 and miR-222 expression increased the growth and tumorigenesis of oral carcinoma cells.," *Journal of oral pathology & medicine : official publication of the International Association of Oral Pathologists and the American Academy of Oral Pathology*, vol. 40, no. 7, pp. 560–6, Aug. 2011, doi: 10.1111/j.1600-0714.2010.01005.x.

[105] C.-X. Xu *et al.*, "MicroRNA miR-214 regulates ovarian cancer cell stemness by targeting p53/Nanog.," *The Journal of biological chemistry*, vol. 287, no. 42, pp. 34970–34978, Oct. 2012, doi: 10.1074/jbc.M112.374611.

[106] J. Xiao, H. Lin, X. Luo, X. Luo, and Z. Wang, "miR-605 joins p53 network to form a p53:miR-605:Mdm2 positive feedback loop in response to stress.," *The EMBO journal*, vol. 30, no. 3, pp. 524–32, Feb. 2011, doi: 10.1038/emboj.2010.347.

[107] N. Zhang, X. Wei, and L. Xu, "miR-150 promotes the proliferation of lung cancer cells by targeting P53.," *FEBS letters*, vol. 587, no. 15, pp. 2346–51, Aug. 2013, doi: 10.1016/j.febslet.2013.05.059.

[108] D.-T. Wang *et al.*, "miR-150, p53 protein and relevant miRNAs consist of a regulatory network in NSCLC tumorigenesis.," *Oncology reports*, vol. 30, no. 1, pp. 492–8, Jul. 2013, doi: 10.3892/or.2013.2453.

[109] Y. Zhang *et al.*, "MicroRNA 125a and its regulation of the p53 tumor suppressor gene.," *FEBS letters*, vol. 583, no. 22, pp. 3725–30, Nov. 2009, doi: 10.1016/j.febslet.2009.10.002.

[110] J. J. Chen, S. X. Liu, M. Z. Chen, and Z. Y. Zhao, "Has-miR-125a and 125b are induced by treatment with cisplatin in nasopharyngeal carcinoma and inhibit apoptosis in a p53-dependent manner by targeting p53 mRNA.," *Molecular medicine reports*, vol. 12, no. 3, pp. 3569–3574, Sep. 2015, doi: 10.3892/mmr.2015.3863.

[111] M. Fabbri *et al.*, "Association of a microRNA/TP53 feedback circuitry with pathogenesis and outcome of B-cell chronic lymphocytic leukemia.," *JAMA*, vol. 305, no. 1, pp. 59–67, Jan. 2011, doi: 10.1001/jama.2010.1919.

[112] Y. Fan *et al.*, "miR-19b promotes tumor growth and metastasis via targeting TP53.," *RNA (New York, N.Y.)*, vol. 20, no. 6, pp. 765–72, Jun. 2014, doi: 10.1261/rna.043026.113.

[113] W. Hu *et al.*, "Negative regulation of tumor suppressor p53 by microRNA miR-504.," *Molecular cell*, vol. 38, no. 5, pp. 689–99, Jun. 2010, doi: 10.1016/j.molcel.2010.05.027.

[114] R. Guo, Y. Wang, W.-Y. Shi, B. Liu, S.-Q. Hou, and L. Liu, "MicroRNA miR-491-5p targeting both TP53 and Bcl-XL induces cell apoptosis in SW1990 pancreatic cancer cells through mitochondria mediated pathway.," *Molecules (Basel, Switzerland)*, vol. 17, no. 12, pp. 14733–47, Dec. 2012, doi: 10.3390/molecules171214733.

[115] K.-W. Hsu *et al.*, "Notch1 pathway-mediated microRNA-151-5p promotes gastric cancer progression.," *Oncotarget*, vol. 7, no. 25, pp. 38036–38051, Jun. 2016, doi: 10.18632/oncotarget.9342.

[116] C. G. Towers *et al.*, "The Six1 oncoprotein downregulates p53 via concomitant regulation of RPL26 and microRNA-27a-3p.," *Nature communications*, vol. 6, p. 10077, Dec. 2015, doi: 10.1038/ncomms10077.

[117] J. Wallace *et al.*, "Genome-Wide CRISPR-Cas9 Screen Identifies MicroRNAs That Regulate Myeloid Leukemia Cell Growth.," *PloS one*, vol. 11, no. 4, p. e0153689, 2016, doi: 10.1371/journal.pone.0153689.

[118] S. R. Chowdhury *et al.*, "Chlamydia preserves the mitochondrial network necessary for replication via microRNA-dependent inhibition of fission.," *The Journal of cell biology*, vol. 216, no. 4, pp. 1071–1089, 2017, doi: 10.1083/jcb.201608063.
[119] L. Chen *et al.*, "MicroRNA-24 increases hepatocellular carcinoma cell metastasis and invasion by targeting p53: miR-24 targeted p53.," *Biomedicine & pharmacotherapy = Biomedecine & pharmacotherapie*, vol. 84, pp. 1113–1118, Dec. 2016, doi: 10.1016/j.biopha.2016.10.051.
[120] X. Tian, G. Zeng, X. Li, Z. Wu, and L. Wang, "Cantharidin inhibits cell proliferation and promotes apoptosis in tongue squamous cell carcinoma through suppression of miR-214 and regulation of p53 and Bcl-2/Bax.," *Oncology reports*, vol. 33, no. 6, pp. 3061–8, Jun. 2015, doi: 10.3892/or.2015.3942.
[121] L.-K. Wang *et al.*, "MicroRNA-133a suppresses multiple oncogenic membrane receptors and cell invasion in non-small cell lung carcinoma.," *PloS one*, vol. 9, no. 5, p. e96765, 2014, doi: 10.1371/journal.pone.0096765.
[122] W. Cui, S. Zhang, C. Shan, L. Zhou, and Z. Zhou, "microRNA-133a regulates the cell cycle and proliferation of breast cancer cells by targeting epidermal growth factor receptor through the EGFR/Akt signaling pathway.," *The FEBS journal*, vol. 280, no. 16, pp. 3962–74, Aug. 2013, doi: 10.1111/febs.12398.
[123] J. Tao *et al.*, "microRNA-133 inhibits cell proliferation, migration and invasion in prostate cancer cells by targeting the epidermal growth factor receptor.," *Oncology reports*, vol. 27, no. 6, pp. 1967–75, Jun. 2012, doi: 10.3892/or.2012.1711.
[124] S.-Y. Lee *et al.*, "The promotion of cardiogenic differentiation of hMSCs by targeting epidermal growth factor receptor using microRNA-133a.," *Biomaterials*, vol. 34, no. 1, pp. 92–9, Jan. 2013, doi: 10.1016/j.biomaterials.2012.09.069.
[125] X. Song, B. Shi, K. Huang, and W. Zhang, "miR-133a inhibits cervical cancer growth by targeting EGFR.," *Oncology reports*, vol. 34, no. 3, pp. 1573–80, Sep. 2015, doi: 10.3892/or.2015.4101.
[126] X. Liu and G. Li, "MicroRNA-133b inhibits proliferation and invasion of ovarian cancer cells through Akt and Erk1/2 inactivation by targeting epidermal growth factor receptor.," *International journal of clinical and experimental pathology*, vol. 8, no. 9, pp. 10605–14, 2015, [Online]. Available: http://www.ncbi.nlm.nih.gov/pubmed/26617770
[127] L. Liu *et al.*, "MicroRNA-133b inhibits the growth of non-small-cell lung cancer by targeting the epidermal growth factor receptor.," *The FEBS journal*, vol. 279, no. 20, pp. 3800–12, Oct. 2012, doi: 10.1111/j.1742-4658.2012.08741.x.
[128] X. Li *et al.*, "Two mature products of MIR-491 coordinate to suppress key cancer hallmarks in glioblastoma.," *Oncogene*, vol. 34, no. 13, pp. 1619–1628, Mar. 2015, doi: 10.1038/onc.2014.98.
[129] W. Chen and Y. Qiu, "Ginsenoside Rh2 Targets EGFR by Up-Regulation of miR-491 to Enhance Anti-tumor Activity in Hepatitis B Virus-Related Hepatocellular Carcinoma.," *Cell biochemistry and biophysics*, vol. 72, no. 2, pp. 325–31, Jun. 2015, doi: 10.1007/s12013-014-0456-9.
[130] D. Yan *et al.*, "MicroRNA-34a inhibits uveal melanoma cell proliferation and migration through downregulation of c-Met.," *Investigative ophthalmology & visual science*, vol. 50, no. 4, pp. 1559–65, Apr. 2009, doi: 10.1167/iovs.08-2681.
[131] J. Yu, D. G. Ryan, S. Getsios, M. Oliveira-Fernandes, A. Fatima, and R. M. Lavker, "MicroRNA-184 antagonizes microRNA-205 to maintain SHIP2 levels in epithelia.," *Proceedings of the National Academy of Sciences of the United States of America*, vol. 105, no. 49, pp. 19300–5, Dec. 2008, doi: 10.1073/pnas.0803992105.
[132] B. Kefas *et al.*, "microRNA-7 inhibits the epidermal growth factor receptor and the Akt pathway and is down-regulated in glioblastoma.," *Cancer research*, vol. 68, no. 10, pp. 3566–72, May 2008, doi: 10.1158/0008-5472.CAN-07-6639.
[133] P. Vasanthan, V. Govindasamy, N. Gnanasegaran, W. Kunasekaran, S. Musa, and N. H. Abu Kasim, "Differential expression of basal microRNAs' patterns in human dental pulp stem cells.," *Journal of cellular and molecular medicine*, vol. 19, no. 3, pp. 566–80, Mar. 2015, doi: 10.1111/jcmm.12381.
[134] S. W. Shahab *et al.*, "The effects of MicroRNA transfections on global patterns of gene expression in ovarian cancer cells are functionally coordinated.," *BMC medical genomics*, vol. 5, p. 33, Aug. 2012, doi: 10.1186/1755-8794-5-33.
[135] X. Zhou *et al.*, "MicroRNA-7 inhibits tumor metastasis and reverses epithelial-mesenchymal transition through AKT/ERK1/2 inactivation by targeting EGFR in epithelial ovarian cancer.," *PloS one*, vol. 9, no. 5, p. e96718, 2014, doi: 10.1371/journal.pone.0096718.

[136] F. C. Kalinowski *et al.*, “Regulation of epidermal growth factor receptor signaling and erlotinib sensitivity in head and neck cancer cells by miR-7.,” *PloS one*, vol. 7, no. 10, p. e47067, 2012, doi: 10.1371/journal.pone.0047067.

[137] A. Barker, K. M. Giles, M. R. Epis, P. M. Zhang, F. Kalinowski, and P. J. Leedman, “Regulation of ErbB receptor signalling in cancer cells by microRNA.,” *Current opinion in pharmacology*, vol. 10, no. 6, pp. 655–61, Dec. 2010, doi: 10.1016/j.coph.2010.08.011.

[138] R. J. Webster, K. M. Giles, K. J. Price, P. M. Zhang, J. S. Mattick, and P. J. Leedman, “Regulation of epidermal growth factor receptor signaling in human cancer cells by microRNA-7.,” *The Journal of biological chemistry*, vol. 284, no. 9, pp. 5731–41, Feb. 2009, doi: 10.1074/jbc.M804280200.

[139] A. C. Midgley, T. Bowen, A. O. Phillips, and R. Steadman, “MicroRNA-7 inhibition rescues age-associated loss of epidermal growth factor receptor and hyaluronan-dependent differentiation in fibroblasts.,” *Aging cell*, vol. 13, no. 2, pp. 235–44, Apr. 2014, doi: 10.1111/acel.12167.

[140] B. Xu *et al.*, “MiR-146a suppresses tumor growth and progression by targeting EGFR pathway and in a p-ERK-dependent manner in castration-resistant prostate cancer.,” *The Prostate*, vol. 72, no. 11, pp. 1171–8, Aug. 2012, doi: 10.1002/pros.22466.

[141] D. R. Hurst, M. D. Edmonds, G. K. Scott, C. C. Benz, K. S. Vaidya, and D. R. Welch, “Breast cancer metastasis suppressor 1 up-regulates miR-146, which suppresses breast cancer metastasis.,” *Cancer research*, vol. 69, no. 4, pp. 1279–83, Feb. 2009, doi: 10.1158/0008-5472.CAN-08-3559.

[142] G. Chen *et al.*, “miR-146a inhibits cell growth, cell migration and induces apoptosis in non-small cell lung cancer cells.,” *PloS one*, vol. 8, no. 3, p. e60317, 2013, doi: 10.1371/journal.pone.0060317.

[143] E. Peta *et al.*, “Down-regulation of microRNA-146a is associated with high-risk human papillomavirus infection and epidermal growth factor receptor overexpression in penile squamous cell carcinoma.,” *Human pathology*, vol. 61, pp. 33–40, 2017, doi: 10.1016/j.humpath.2016.10.019.

[144] S. Ali *et al.*, “Deregulation of miR-146a expression in a mouse model of pancreatic cancer affecting EGFR signaling.,” *Cancer letters*, vol. 351, no. 1, pp. 134–42, Aug. 2014, doi: 10.1016/j.canlet.2014.05.013.

[145] R. Kogo, K. Mimori, F. Tanaka, S. Komune, and M. Mori, “Clinical significance of miR-146a in gastric cancer cases.,” *Clinical cancer research : an official journal of the American Association for Cancer Research*, vol. 17, no. 13, pp. 4277–84, Jul. 2011, doi: 10.1158/1078-0432.CCR-10-2866.

[146] J. Cai, L. Xu, Z. Cai, J. Wang, B. Zhou, and H. Hu, “MicroRNA-146b-5p inhibits the growth of gallbladder carcinoma by targeting epidermal growth factor receptor.,” *Molecular medicine reports*, vol. 12, no. 1, pp. 1549–55, Jul. 2015, doi: 10.3892/mmr.2015.3461.

[147] Y. Li *et al.*, “miR-146a suppresses invasion of pancreatic cancer cells.,” *Cancer research*, vol. 70, no. 4, pp. 1486–95, Feb. 2010, doi: 10.1158/0008-5472.CAN-09-2792.

[148] X. Sun *et al.*, “miR-7 reverses the resistance to BRAFi in melanoma by targeting EGFR/IGF-1R/CRAF and inhibiting the MAPK and PI3K/AKT signaling pathways.,” *Oncotarget*, vol. 7, no. 33, pp. 53558–53570, Aug. 2016, doi: 10.18632/oncotarget.10669.

[149] M. Talekar *et al.*, “Combination wt-p53 and MicroRNA-125b Transfection in a Genetically Engineered Lung Cancer Model Using Dual CD44/EGFR-targeting Nanoparticles.,” *Molecular therapy : the journal of the American Society of Gene Therapy*, vol. 24, no. 4, pp. 759–69, Apr. 2016, doi: 10.1038/mt.2015.225.

[150] X. Zhou *et al.*, “Downregulation of miR-21 inhibits EGFR pathway and suppresses the growth of human glioblastoma cells independent of PTEN status.,” *Laboratory investigation; a journal of technical methods and pathology*, vol. 90, no. 2, pp. 144–55, Feb. 2010, doi: 10.1038/labinvest.2009.126.

[151] T. Chiyomaru *et al.*, “Genistein up-regulates tumor suppressor microRNA-574-3p in prostate cancer.,” *PloS one*, vol. 8, no. 3, p. e58929, 2013, doi: 10.1371/journal.pone.0058929.

[152] S. Ohno *et al.*, “Systemically injected exosomes targeted to EGFR deliver antitumor microRNA to breast cancer cells.,” *Molecular therapy : the journal of the American Society of Gene Therapy*, vol. 21, no. 1, pp. 185–91, Jan. 2013, doi: 10.1038/mt.2012.180.

[153] Y. Li *et al.*, “MicroRNA-27a functions as a tumor suppressor in renal cell carcinoma by targeting epidermal growth factor receptor.,” *Oncology letters*, vol. 11, no. 6, pp. 4217–4223, Jun. 2016, doi: 10.3892/ol.2016.4500.

[154] L. K. Mathew *et al.*, “miR-218 opposes a critical RTK-HIF pathway in mesenchymal glioblastoma.,” *Proceedings of the National Academy of Sciences of the United States of America*, vol. 111, no. 1, pp. 291–6, Jan. 2014, doi: 10.1073/pnas.1314341111.

[155] K. Zhu *et al.*, “Tumor-suppressive miR-218-5p inhibits cancer cell proliferation and migration via EGFR in non-small cell lung cancer.,” *Oncotarget*, vol. 7, no. 19, pp. 28075–85, May 2016, doi: 10.18632/oncotarget.8576.

[156] B. Xu *et al.*, "hsa-miR-135a-1 inhibits prostate cancer cell growth and migration by targeting EGFR.," *Tumour biology : the journal of the International Society for Oncodevelopmental Biology and Medicine*, vol. 37, no. 10, pp. 14141–14151, Oct. 2016, doi: 10.1007/s13277-016-5196-6.

[157] M. L. Ufkin, S. Peterson, X. Yang, H. Driscoll, C. Duarte, and P. Sathyanarayana, "miR-125a regulates cell cycle, proliferation, and apoptosis by targeting the ErbB pathway in acute myeloid leukemia.," *Leukemia research*, vol. 38, no. 3, pp. 402–10, Mar. 2014, doi: 10.1016/j.leukres.2013.12.021.

[158] W. Wang, B. Cheng, L. Miao, Y. Mei, and M. Wu, "Mutant p53-R273H gains new function in sustained activation of EGFR signaling via suppressing miR-27a expression.," *Cell death & disease*, vol. 4, p. e574, Apr. 2013, doi: 10.1038/cddis.2013.97.

[159] M. Acunzo *et al.*, "Cross-talk between MET and EGFR in non-small cell lung cancer involves miR-27a and Sprouty2.," *Proceedings of the National Academy of Sciences of the United States of America*, vol. 110, no. 21, pp. 8573–8, May 2013, doi: 10.1073/pnas.1302107110.

[160] W. C. S. Cho, A. S. C. Chow, and J. S. K. Au, "MiR-145 inhibits cell proliferation of human lung adenocarcinoma by targeting EGFR and NUDT1.," *RNA biology*, vol. 8, no. 1, pp. 125–31, doi: 10.4161/rna.8.1.14259.

[161] A. R. Glover *et al.*, "MicroRNA-7 as a tumor suppressor and novel therapeutic for adrenocortical carcinoma.," *Oncotarget*, vol. 6, no. 34, pp. 36675–88, Nov. 2015, doi: 10.18632/oncotarget.5383.

[162] T. Chiyomaru *et al.*, "Dual regulation of receptor tyrosine kinase genes EGFR and c-Met by the tumor-suppressive microRNA-23b/27b cluster in bladder cancer.," *International journal of oncology*, vol. 46, no. 2, pp. 487–96, Feb. 2015, doi: 10.3892/ijo.2014.2752.

[163] L. Wang *et al.*, "MicroRNA-302b suppresses cell proliferation by targeting EGFR in human hepatocellular carcinoma SMMC-7721 cells.," *BMC cancer*, vol. 13, p. 448, Oct. 2013, doi: 10.1186/1471-2407-13-448.
